# Single-cell RNA sequencing reveals the heterogeneity of liver-resident immune cells in human

**DOI:** 10.1038/s41421-020-0157-z

**Published:** 2020-04-28

**Authors:** Juanjuan Zhao, Shuye Zhang, Yang Liu, Xiaomeng He, Mengmeng Qu, Gang Xu, Hongbo Wang, Man Huang, Jing Pan, Zhenwen Liu, Zhiwei Li, Lei Liu, Zheng Zhang

**Affiliations:** 1 Institute of Hepatology, National Clinical Research Center for Infectious Disease, Shenzhen Third People’s Hospital, Shenzhen, Guangdong 518112 China; 2 The Second Affiliated Hospital, School of Medicine, Southern University of Science and Technology, Shenzhen, Guangdong 518112 China; 3 Shanghai Public Health Clinical Center and Institute of Biomedical Sciences, Fudan University, Shanghai, 201058 China; 4 Research Center for Clinical & Translational Medicine, Fifth Medical Center for General Hospital of PLA, Beijing, 100039 China; 5 Research Center for Liver Transplantation, Fifth Medical Center for General Hospital of PLA, Beijing, 100039 China; 6 Deparment for Liver Transplantation, Shenzhen Third People’s Hospital, Shenzhen, Guangdong 518112 China; 7 Key Laboratory of Immunology, Sino-French Hoffmann Institute, School of Basic Medical Sciences; Guangdong Provincial Key Laboratory of Allergy & Clinical Immunology, The Second Affiliated Hospital, Guangzhou Medical University, Guangzhou, 511436 China

**Keywords:** Immunology, Cell biology

## Abstract

The liver plays a critical role in both immune defense and tolerance in the body. The liver-resident immune cells (LrICs) determine the immune properties, but the unique composition and heterogeneity of these cells are incompletely understood. Here, we dissect the diversity of LrICs by a comprehensive transcriptomic profiling using the unbiased single-cell RNA-sequencing (scRNA-seq). A total of 70, 706 of CD45^+^ immune cells from the paired liver perfusion, spleen and peripheral blood as references were profiled. We identified more than 30 discrete cell populations comprising 13 of T and NK cell, 7 of B cell, 4 of plasma cell, and 8 of myeloid cell subsets in human liver and donor-paired spleen and blood, and characterized their tissue distribution, gene expression and functional modules. Especially, four of CXCR6^+^ T and NK cell subsets were found to be present preferentially in the liver, where they manifested heterogeneity, distinct function and prominent homeostatic proliferation. We propose a universal category system of T and NK cells based on distinct chemokine receptors, confirmed subsequently by phenotype, transcriptional factors and functionality. We also identified adaptive changes by the spleen and liver-derived monocyte and macrophage populations. Finally, we give a global glimpse on B cell and plasma cell subsets in human spleen and liver. We, therefore, reveal the heterogeneity and functional diversity of LrICs in human. This study presents comprehensively the landscape of LrICs and will enable further study on their roles in various human diseases.

## Introduction

The liver is the largest human solid organ and sits between the digestive and circulatory systems. Essential functions of the liver, include protein synthesis and metabolism of amino acids, carbohydrates, lipids and vitamins. Immunologically, the liver is not only responsible for detecting exogenous pathogens and antigens in systemic circulation, but also is tasked with facilitating immune tolerance which protects the host from antigenic overload of dietary components, drugs, and bacterial products originating in the gut^1^. The underlying mechanisms maintaining these dual immune properties of liver are yet to be determined. Dissecting the unique milieu and function of hepatic immune cells, which are significantly different from those present in immune organs (e.g., spleen, lymph nodes, and bone marrow), will be essential for understanding liver-specific immunological properties and immune-mediated events implicated in liver diseases^2,3^. Liver immune cells are heterogeneous and consist of multiple subsets with various immune functions, including not only of conventional T cells, B cells, natural killer (NK) cells, and monocytes^4^. Recent reports identify the existence of LrT cells^5^, LrNK cells^6,7^ and macrophage^8^, which display notable differences compared to those in circulation^9^. These liver-resident immune cells (LrICs) are also affected by tissue microenvironment in which regulatory modules are suspected to play crucial roles in various liver diseases^10^. However, these studies focused on particular cell subsets or limited numbers of markers using classical flow cytometry, and comprehensive characterization of immune cellular compartments and heterogeneity in the human liver has not been well performed. A reference map of the healthy human liver immune cell landscape at single-cell resolution is critical to understanding immune pathogenesis and treatment of liver disease, but has not previously been established. The recent development of unbiased single-cell RNA sequencing (scRNA-seq) technology is well suited to resolve immune cellular complexity and heterogeneity, identify new cell subsets and DEGs and pathways, and delineate underlying cell lineage relationships^11^. Although scRNA-seq has been used to dissect the whole liver in human^12,13^ and mouse^14^, peripheral blood mononuclear cells (PBMCs)^15^, NK cells^16^, dendritic cells (DCs)^17^, and innate lymphoid cells (ILCs)^18^, it has not yet been used to assess the whole scenario of liver immune cells in healthy donors. Previous studies were also limited by the total immune cell numbers analyzed, and no matched blood cells for comparison. Thus, previous studies have not succeeded in depicting a high-resolution liver immune cell atlas and LrICs could not be faithfully confirmed. The spleen is the largest immune organ. Anatomically, spleen vein blood is collected into the liver via the portal vein. Therefore, paired spleen and blood are optimal controls for the characterization of LrICs. Here, we use scRNA-seq to provide the first unbiased and comprehensive atlas of intrahepatic CD45^+^ immune cells in relation to paired spleen and PBMCs from liver transplant donors. This atlas will serve as a crucial resource to enable further study on the role of various immune subsets in the human liver and spleen as well as various liver diseases.

## Results

### ScRNA-seq maps distinct immune cell populations in the human liver

**Fig. 1:**
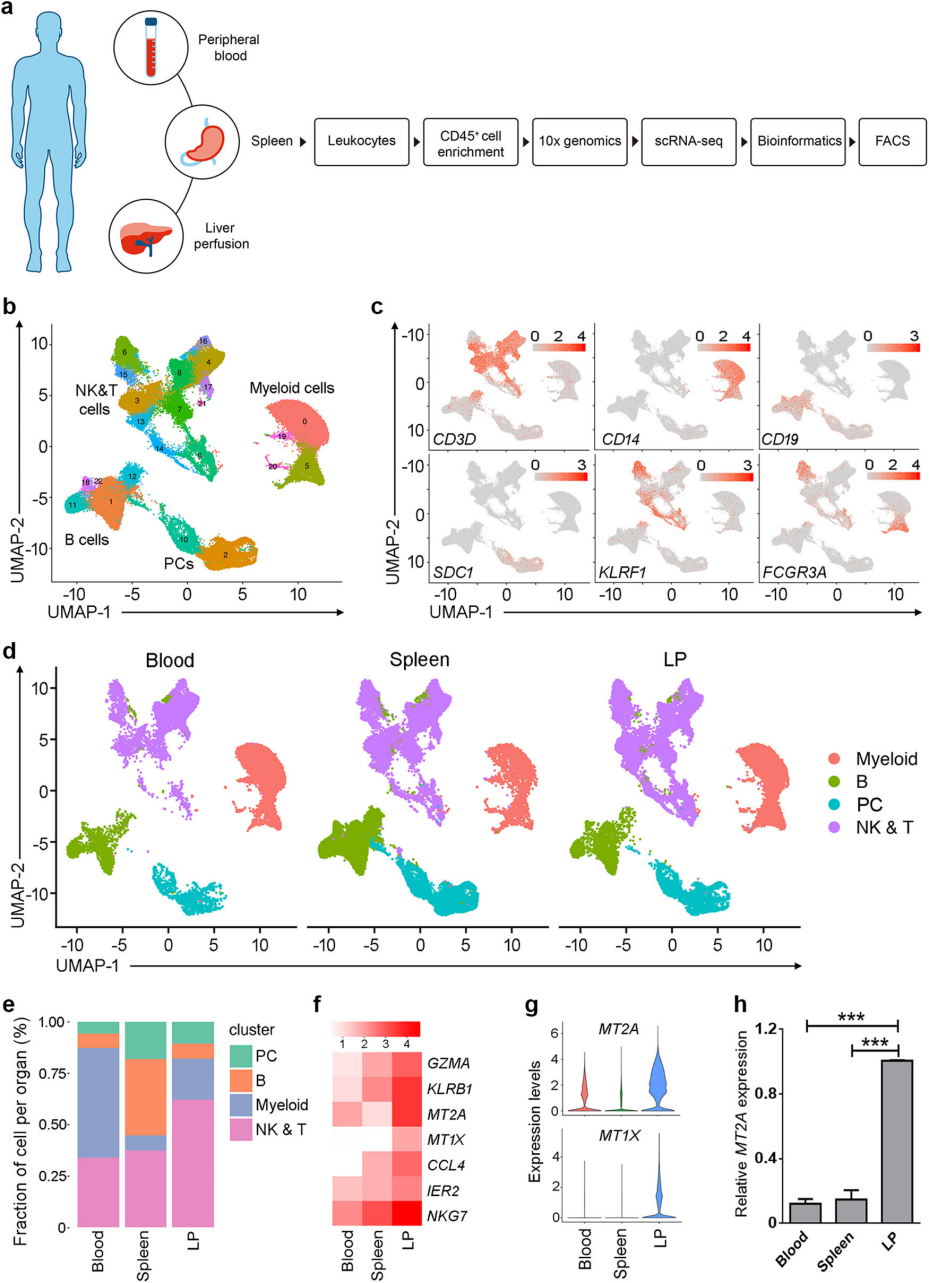
Single-cell transcriptomics identify distinct immune cell populations and specific markers in the human liver. **a** Workflow of the single cell isolation and analysis using 10× Genomics platform. **b** UMAP plot of the immune cells showing 23 clusters belonging to 4 major groups. **c** The classical markers indicating group identities. **d** The UMAP plots of the immune cells by their tissue source. **e** The proportion of major immune subsets in human blood, spleen, and liver perfusion (LP). **f** Relative expression for seven genes identified as unique to liver-derived immune cells. **g** Violin plots showing the expression of *MT1X* and *MT2A* at the single cell level. **h** Real-time PCR confirmed the specific expression of *MT2A* in liver-derived immune cells (*n* = 5). ***P < 0.001, data are represented as mean ± SEM. LP liver perfusion.

We performed scRNA-seq analysis of CD45^+^ cells from three donors with normal histology (Supplementary Fig. S1) using paired liver perfusion (LP), spleen, and PB (Supplementary Table S1 and Supplementary Fig. S2). Briefly, live CD45^+^ immune cells were isolated from fresh samples by magnetic selection and single-cell transcriptomes were obtained using the 10× Genomics platform (Fig. 1a). The final dataset comprised 70, 706 single-cell transcriptomes merged from triplicate blood, spleen, and LP samples (Supplementary Table S2). The resulting single-cell profiles consistently passed stringent high-quality filtering (Supplementary Fig. S2), yielding a mean of 61, 000 reads/cell with median gene and unique molecular identifier (UMI) counts of 1, 304 and 4, 213, respectively (Supplementary Table S2).

We applied principal component analysis (PCA) using the top variable genes ranked by their normalized dispersion as previously reported^15^. We then used the first 50 PC loadings as input to Uniform Manifold Approximation and Projection (UMAP) for visualization and clustered cells using a graph-based method. We observed 23 distinct cell clusters (C) composed of 4 major cell subtypes, including NK & T, B, myeloid and plasma cells (PC) (Fig. 1b), each identified by their unique signature genes *CD3D* (T cell marker), *KLRF1* (NKp80, NK marker), *CD19* (B cell marker), *SDC1* (CD138, PC marker), *CD14* (monocyte marker) and *FCGR3A* (CD16), respectively (Fig. 1c). We confirmed that the data integration removed residual batch effect and enabled excellent reproducibility across different donors (Supplementary Fig. S3a). Indeed, every individual cluster consisted of considerable proportion of cells from each donor (Supplementary Fig. S3b).

Thus, the unbiased scRNA-seq data allowed comparison of the distribution of cell compartments among LP, spleen, and blood. It was apparent that higher proportions of T and NK cells in the LP, B cells in the spleen and myeloid cells in blood (Fig. 1d, e). This data suggest that the fine structure of immune cell compartments differs significantly in these tissues and confirm a faithful recapitulation of the overall immune cell atlas.

We next sought to identify specific signatures for liver-derived lymphocytes. We screened for liver-specific genes in four major immune subsets compared to those from spleen and blood (Supplementary Fig. S3c). Seven genes are highly expressed by liver-derived subsets (Supplementary Fig. S3d). Notably, metallothionein (MT, a metal-binding protein regulating zinc and copper homeostasis) gene expression was significantly higher in the LP than that in the spleen and blood (Fig. 1f). Among MTs, *MT2A* and *MT1X* were the most highly expressed (Fig. 1g). We confirmed these findings at the mRNA level by qRT-PCR (Fig. 1h). We also found that cytotoxic molecules *GZMA* and *NKG7*, chemokine *CCL4*, and *KLRB1* (CD161) and *IER2* were highly expressed in liver-derived lymphocytes (Supplementary Fig. S3d). These molecules specially related to liver-derived cell subsets may shape the unique human liver immune microenvironment.

### Identification of LrT and LrNK cells

**Fig. 2:**
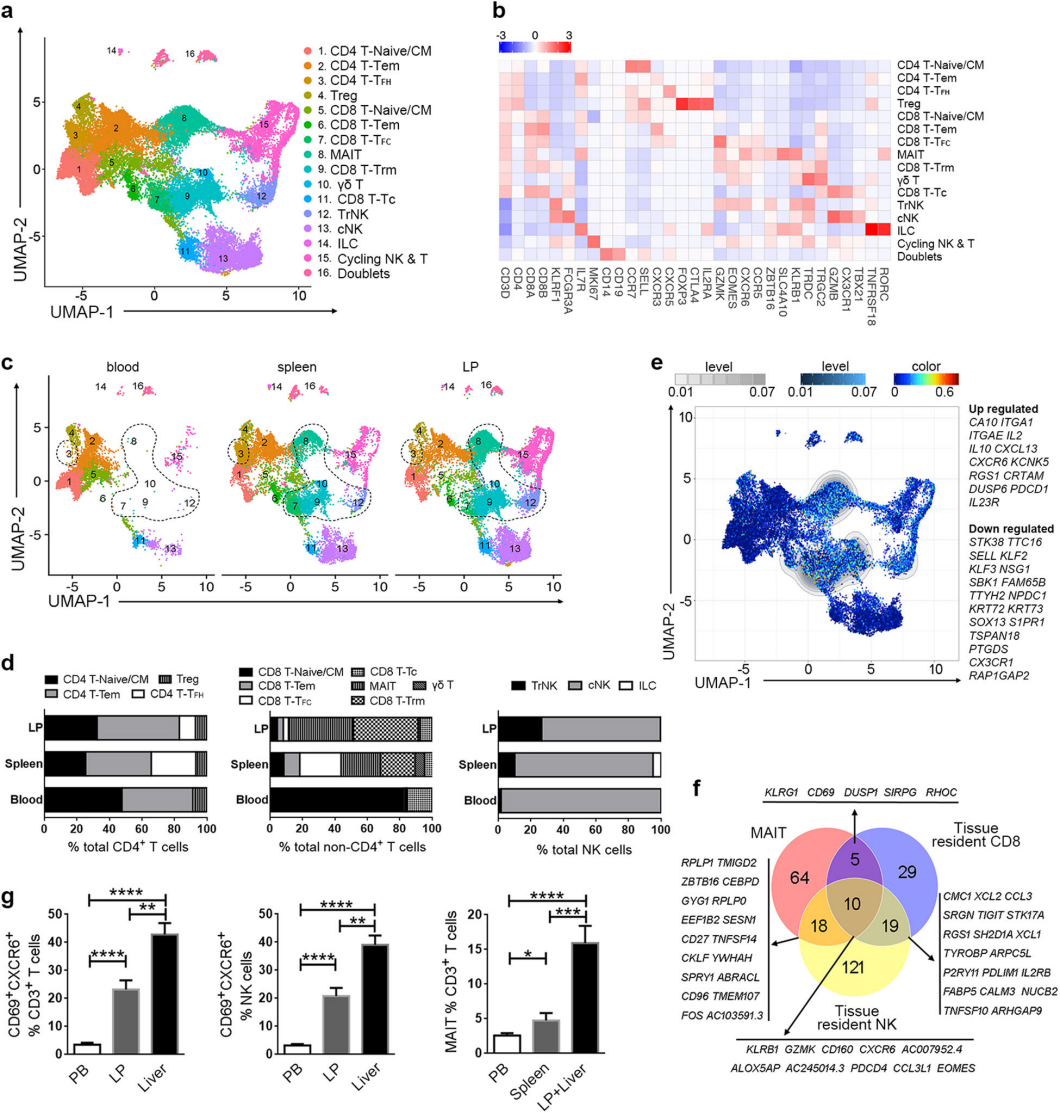
Characterization of tissue-resident T and NK cells in human spleen and liver. **a** UMAP analysis of human T and NK cells showing 16 clusters. **b** Heatmap showing crucial marker genes among 16T and NK cell subsets. Marker gene names are labeled at the bottom. **c** The UMAP plots of the T and NK cells by their tissue source. The dotted circle indicated the tissue resident populations. **d** The proportion of CD4^+^ T cell, CD8^+^ T cell and NK cell subsets in human blood, spleen and LP. **e** Previously reported Trm core signatures, composed of 31 differentially expressed genes between CD69^+^ and CD69^−^ memory T cells, identifies MAIT, Trm, γδT and TrNK cells. **f** Venn plot indicating the distribution of specific expressed gene counts for each liver-resident T and NK cell subset. Genes co-expressed by any two liver-resident cell subsets were listed. **g** Flow cytometric data of CD69^+^CXCR6^+^ Trm, TrNK, and MAIT cells from peripheral blood (*n* = 19), liver perfusion (*n* = 10), and liver (*n* = 20), respectively.

Although this combined analysis provided an overall picture of the immune cell atlas, the large data size might hinder further dissection of the subclusters of each major immune cell subset in detail. Therefore, we first comprehensively dissected the T and NK cells based on variably expressed genes. Four of CD4^+^ T cell, 7 of CD8^+^ T cell, 2 of NK cell, 1 of ILCs and 1 of proliferative cell subsets were identified and visualized using UMAP (Fig. 2a) basing on the typical marker expressions and their unique distributions (Fig. 2b; Supplementary Fig. S4a, Table S3). C1 and C5 identify the naïve/central memory (CM) CD4^+^ and CD8^+^ T cells. C2 and C6 identifies circulating CD4^+^ and CD8^+^ T memory (Tem) cells. C3 and C7 identify the follicular CD4^+^ (T_FH_) and CD8^+^ T (T_FC_) cells. C4 identifies the regulatory T (Treg) cells. C8–C10 represents the tissue-resident T-cell subset. C8 identifies mucosal-associated invariant T cells (MAIT) cells, C9 for CD8^+^ tissue-resident memory T (Trm) cells, C10 for γδ T cells, C11 for CD8^+^ T cell expressing cytotoxic markers (GZMB^+^ CD8^+^ Tc). C12 is the tissue-resident NK (TrNK) subset, while C13 is the conventional NK subset expressing cytotoxic markers (GZMB^+^ cNK). C14 identifies ILCs. C15 identifies replicating T and NK cell subsets. Comparing blood, spleen and liver facilitated the dissection of tissue distribution patterns by various T and NK cell subsets (Fig. 2c). We identified that C3 (T_FH_) and C7 (T_FC_) preferentially resided in spleen representing the follicular CD4^+^ and CD8^+^ T-cell populations, respectively, while C8 (MAIT), C9 (Trm), C10 (γδ T), and C12 (TrNK) resided in human liver as the tissue resident cell populations (Fig. 2c, d).

To search for underlying core signatures shared by Trm and TrNK cells. We first validated the previously reported core signatures of CD69^+^ Trm cells in our dataset^19^. This 31 core genes composed signatures, combining two gene sets (upregulated and downregulated genes of CD69^+^ compared with CD69^−^ memory T cells), was scored for each single cell and visualized in the UMAP by Single-Cell Signature Explorer^20^. We found that this core signature perfectly matched the MAIT, Trm, γδ T, and TrNK cells (Fig. 2e). Next, we sought unique gene sets expressed by Trm and TrNK cells through its comparison with liver-derived Tem and GZMB^+^ CD8^+^ Tc cells (Supplementary Fig. S4b, Table S4). In total, 63 genes were highly expressed in Trm cells including chemokines (*XCL2*, *CCL3*, and *CCL4*), cell adhesion molecules (*ICAM1*, *VCAM1*, and *ITGAD*), transcriptional factors (*EOMES* and *IKZF2*) and others. Similarly, unique genes in MAIT cells included MAIT-specific genes (*SLC4A10* and *KLRB1*), chemokine receptors (*CXCR6*, *CCR5*, and *CCR6*), transcriptional factors (*IKZF2* and *ZBTB16*). The specific genes by TrNK cells were also comparable to MAIT and CD8^+^ Trm cells when compared to liver GZMB^+^ cNK cells. This comparative analysis revealed the core genes of TrNK and Trm cells, including *KLRB1*, *GZMK*, *CD160*, *CXCR6*, *EMOES*, etc. (Fig. 2f; Supplementary Table S5). Finally, we validated that one of these unique surface marker CXCR6 was co-expressed with CD69 (one classical tissue-resident marker) (Supplementary Fig. S5a) but lacked of CD62L (Supplementary Fig. S5b, c) by flow cytometry, and further demonstrated that CXCR6^+^CD69^+^ Trm, TrNK, and MAIT cells were enriched in the human liver and spleen as compared to blood (Fig. 2g).

### Characterization of cycling liver-resident T and NK cells

**Fig. 3:**
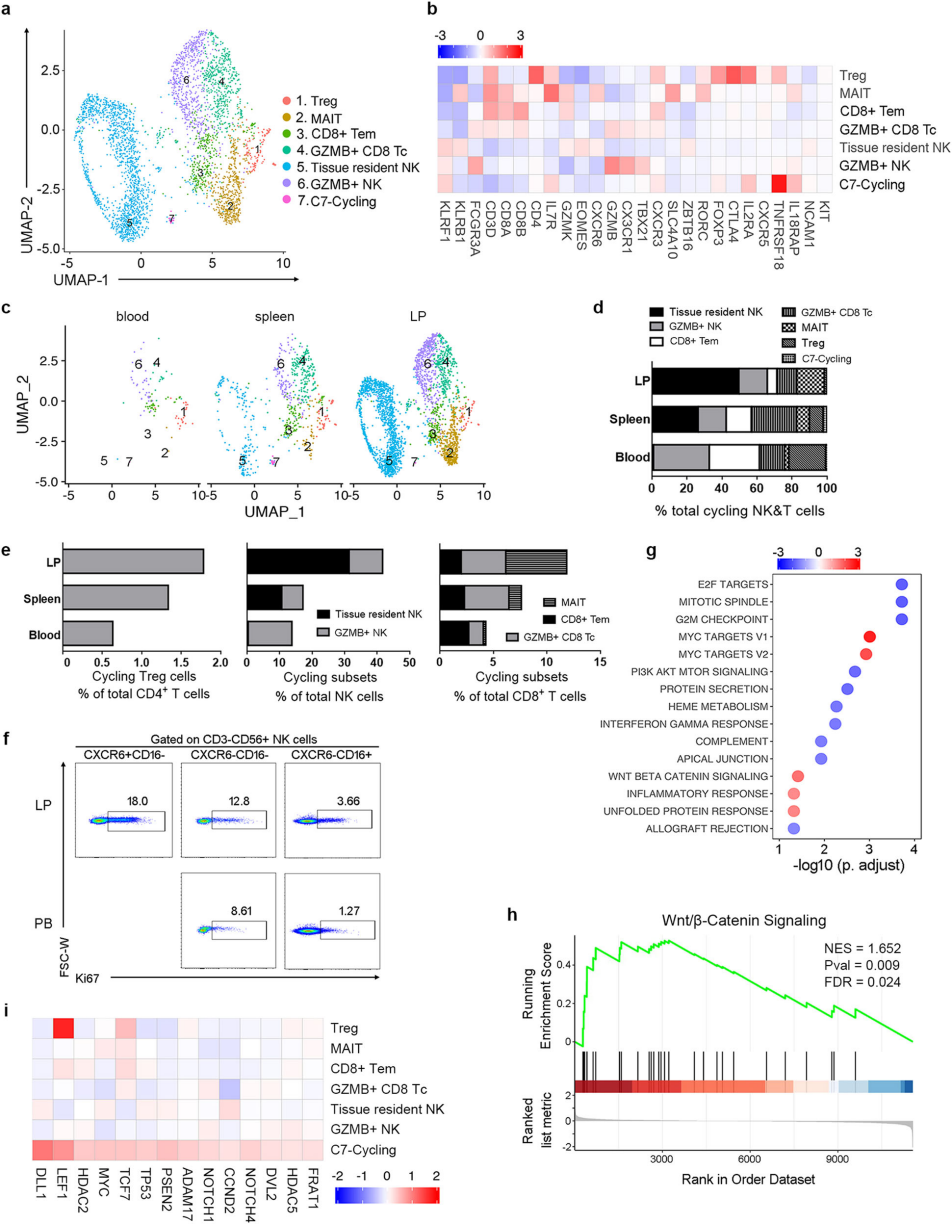
Characterization of cycling T and NK cells from human spleen and liver by scRNA-seq. **a** UMAP plot of cycling T and NK cells showing a seven-cluster distribution. **b** Heatmap of selected markers among seven different cycling T and NK cell subsets. **c** UMAP plot of cycling NK&T cell subsets indicated by tissue source. **d** The proportion of seven cycling T and NK subsets in human blood, spleen, and LP. **e** The proportion of each cycling NK&T subset except the cycling-new cluster among total CD4^+^ T cells, CD8^+^ T cells and NK cells in human blood, spleen, and LP. **f** Representative dot plots indicating the ki67 expression in CXCR6^+^CD16^−^, CXCR6^−^CD16^−^ and CXCR6^−^CD16^+^ NK cell subsets from paired peripheral blood and LP (*n* = 3), respectively. The number indicated the proportion of ki67^+^ cells among each NK subsets. **g** GESA analysis of the C7-cycling subset showed enriched pathway from hallmark gene sets. **h** The GSEA plot of Wnt-beta-Catenin pathway enriched in the C7-cycling subset. **i** The heatmap showing specific expression of Wnt-beta-Catenin pathway genes by C7-cycling subset.

As shown Fig. 2a, C15 was identified as cycling T and NK cells that expressed replicating markers such as *MKI67* in comparison with non-cycling T and NK cells (Supplementary Fig. S4a). Among cycling T and NK cells, seven cycling subclusters could be identified (Fig. 3a) basing on typical marker expression (Fig. 3b; Supplementary Fig. S6, Table S6). C1–C6 identifies the cycling Treg cells, MAIT cells, CD8^+^ Tem cells, GZMB^+^ CD8^+^ Tc cells, TrNK and GZMB^+^ cNK cells, respectively. Cells in C7 belong to a lymphoid subset with NK cell markers, such as *NCAM1*, *KLRF1*, and early differentiation markers, including *CCR7*, *SELL*, *IL7R*, *IL18RAP*, and *TNFRSF18* (Fig. 3b; Supplementary Fig. S6).

Tissue-residence patterns of cycling T and NK cell subsets in blood, spleen, and LP was shown in UMAP plot (Fig. 3c). Peripheral cycling T and NK cells did not contain TrNK and MAIT cells. By contrast, liver and spleen cycling T and NK cells contained more TrNK and MAIT (Fig. 3d). In addition, the proportion of cycling TrNK, Treg, and MAIT cells within NK, CD4^+^ T, and CD8^+^ T cells was higher in the LP compared to blood and spleen (Fig. 3e). Furthermore, FACS data confirmed that hepatic CXCR6^+^CD16^−^ TrNK cells displayed higher levels of ki67 expression than that of CXCR6^−^CD16^+^ NK cells, suggesting that LrNK cells were characterized by more robustly proliferative status in human (Fig. 3f). These findings highlight the impact of tissue residence on T and NK cell homeostasis.

To characterize the feature of C7-cycling, we analyzed its transcriptomic feature using GSEA on the Hallmark gene sets (Fig. 3g). There is a significant enrichment of genes from Wnt-beta-Catenin, Myc target, unfolded protein response, and inflammatory response pathways. We identified that many genes involved in Wnt-beta-Catenin pathways, Myc targets were significantly upregulated in this particular cell cluster (Fig. 3h, i; Supplementary Fig. S7). Together with the expression of NK cell markers and early differentiation markers, C7 likely represents a NK lineage or a lymphoid progenitor cell population^21^.

### A novel classification system for T and NK cells based on chemokine receptors

**Fig. 4:**
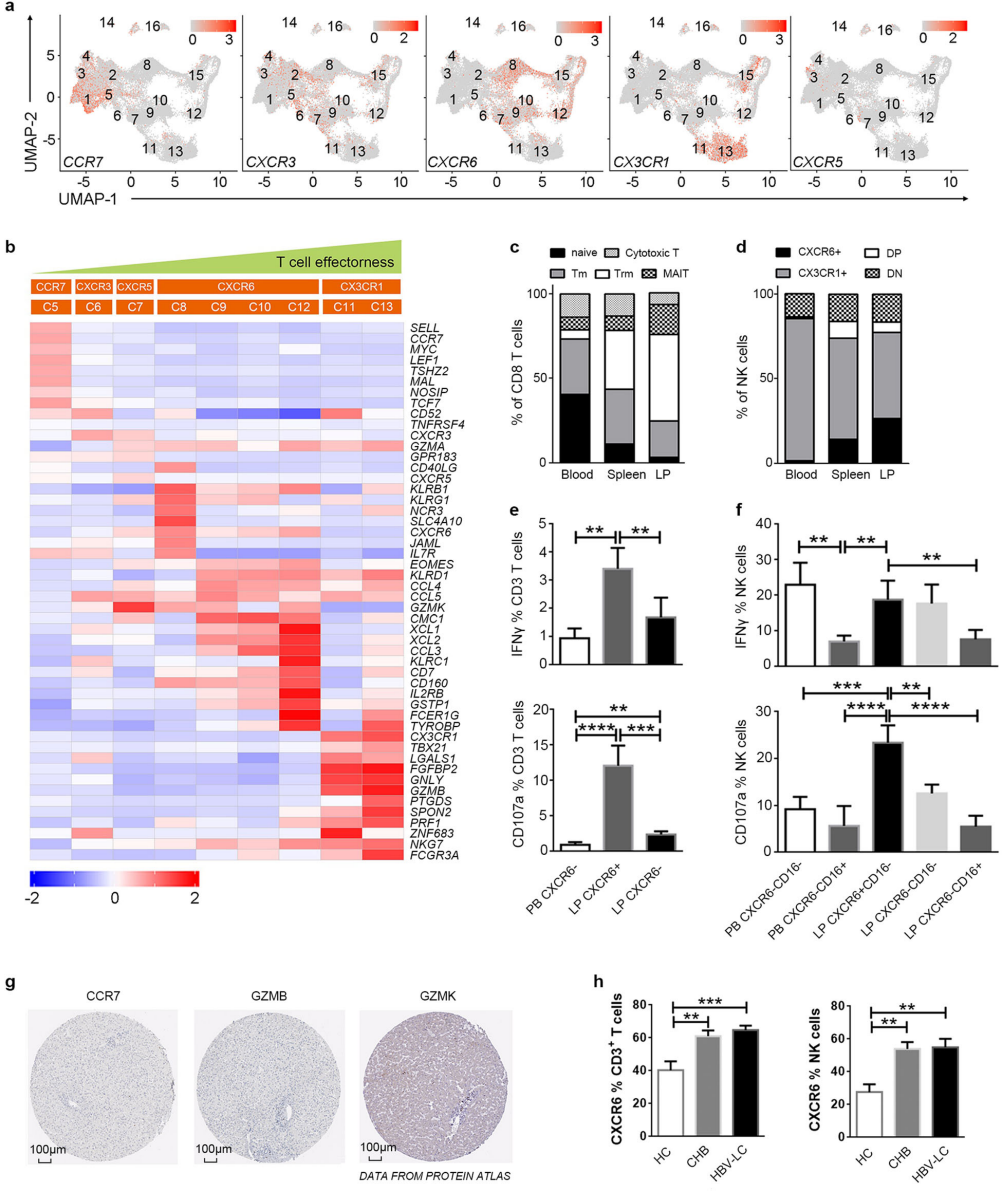
Novel classification of T and NK cells based on specific chemokine receptor expression. **a** UMAP plots showing the distribution of chemokine receptors CCR7, CXCR3, CXCR6, CX3CR1, and CXCR5 matching with various T and NK cell subsets. **b** The heatmap indicating the expression of specific genes by chemokine receptors-defined NK & T cell clusters. Chemokine receptors expression correlates with the differentiation, effectorness and distribution patterns of CD8^+^ T and NK cell subsets. **c** The pool data by flow cytometry indicating the proportion of CCR7^+^ naive/CM T cells, CXCR3^+^ Tm, CXCR6^+^ Trm and CX3CR1^+^ Tc cells among CD8 T cells from peripheral blood (*n* = 13), liver perfusion (*n* = 5), and liver (*n* = 9), respectively. **d** The pool data by flow cytometry indicating the proportion of CXCR6^+^CX3CR1^−^ TrNK, CXCR6^−^CX3CR1^+^ NK cells, and CXCR6^+^CX3CR1^+^ double-positive (DP) and CXCR6^−^CX3CR1^−^ double-negative (DN) cells among CD3^-^CD56^+^ total NK cells from peripheral blood (*n* = 13), liver perfusion (*n* = 5), and liver (*n* = 9), respectively. **e** Summary data indicating CD107a and IFN-γ production by CXCR6^+^ Trm from LP as compared to those from CXCR6^−^ T cells from LP (*n* = 11) and blood (*n* = 12) in responses to IL-12/IL-18 stimulations, respectively. **f** Summary data indicating CD107a and IFN-γ production by CXCR6^+^CD16^−^ TrNK cells from LP as compared to other NK cell subsets from LP (*n* = 11) and blood (*n* = 12) in responses to IL-12/IL-18 stimulations, respectively. **P < 0.01, ***P < 0.001, ****P < 0.0001, data are represented as mean ± SEM. PB peripheral blood, LP liver perfusion. **g** Immuno-histochemistry staining of CCR7, GZMK, and GZMB in the human liver by histological data from Protein Atlas, which represent naïve cells, tissue-resident cells and cNK cells, respectively. **h** The proportion of CXCR6 + cells among total T cells and NK cells from the liver biopsy in HC (*n* = 8), CHB patients (*n* = 12), and HBV-associated liver cirrhosis patients (*n* = 10). *P < 0.05, **P < 0.01, data are represented as mean ± SEM.

Interestingly, we found that several key chemokine receptors (*CCR7*, *CXCR3*, *CXCR5*, *CXCR6*, and *CX3CR1*) have potentials to effectively identify most of T cell and NK cell subsets (Fig. 4a). For example, *CCR7* is uniquely expressed by naïve/CM CD4^+^ (C1) and CD8^+^ T cells (C5). *CXCR3* is expressed by memory CD4^+^ (C2) and CD8^+^ T cells (C6). *CXCR5* is expressed by follicular CD4^+^ (C3) and CD8^+^ T cells (C7). *CXCR6* is preferentially expressed by MAIT (C8), CD8^+^ Trm (C9), γδ T (C10) and TrNK cells (C12). *CX3CR1* is uniquely expressed by GZMB^+^ CD8^+^ Tc (C11) and cNK cells (C13). The chemokine receptor expression patterns of NK & T cells correlate with their distinct tissue distribution. We observed liver- and spleen-derived NK & T cells expressed higher levels of *CXCR6*, while blood T cells mainly expressed *CCR7* (Fig. 2d; Supplementary Fig. S8). Importantly, this particular category system for T and NK cells based on these five chemokine receptors correlate with the effectorness differentiation scheme of T and NK cells (Fig. 4b). CCR7 defines the naive/CM state identified by *MYC*, *LEF1*, *TSHZ2*, *MAL*, *NOSIP*, and *TCF7* expression. CXCR3/CXCR5 defines a more intermediate differentiation state identified by expressing intermediate levels of effector molecules. CXCR6 defines the tissue resident lineages identified by the aforementioned resident core signature molecules, including *EOMES*, *GZMK*, etc., while the CX3CR1 defined the terminal differentiation state with co-expression of many cytotoxic molecules, including *FGFBP2*, *GZMB*, *GNLY*, *SPON2*, *PRF1*, etc.

We further confirmed the chemokine receptor-based categorization of CD8^+^ T and NK cells using flow cytometry (Supplementary Fig. S9a). Through excluding MAIT cells based on TCR-Vα7.2 and CD161, the remaining CD8^+^ T cells contain CCR7^+^ naïve T (Tnaïve) cells and CCR7^−^ memory cells, the latter can be further divided into CXCR6^+^CX3CR1^−^ Trm, CXCR6^−^CX3CR1^+^ Tc and CXCR6^−^CX3CR1^−^ Tem cells. The pool data also confirmed that CXCR6^+^ CD8^+^ T cells and MAIT cells are enriched in the LP, while Tnaïve cells and Tem cells are distributed in the blood (Fig. 4c). Importantly, these four populations of CD8^+^ T cells differentially express various transcriptional factors (TFs) (Supplementary Fig. S9a). Tnaïve cells lack all four TFs detected, while other memory T cells express EOMES. Trm preferentially expresses EMOES, PLZF, and Helios, while CX3CR1^+^ Tc cells preferentially expresses T-bet. Similarly, CXCR6^+^ NK cells which mainly reside in the liver (Fig. 4d) express high levels of EOMES and PLZF, while CX3CR1^+^ NK cells only express T-bet (Supplementary Fig. S9b). Functionally, we found that CXCR6^+^ LrT cells and CXCR6^+^CD16^−^ LrNK cells displayed more capacity to produce IFN-γ and CD107a in response to IL-12/IL-18 stimulation in vitro as compared to those from PB- and liver-derived CXCR6^−^CD16^+^ T & NK cells (Fig. 4e, f; Supplementary Fig. S9c, d). Histologically, as shown by data from Protein atlas (Fig. 4g), very few number of CCR7^+^ cells (naive/CM T cells) are present in the liver, and GZMB^+^ cells (Tc and NK cells) are concentrated in the portal area, while GZMK^+^ (Trm and TrNK) cells scattered evenly in the liver sinusoids and their numbers are much more than GZMB^+^ cells. We also found that HBV-infected livers contained more infiltration of CXCR6^+^ LrT and LrNK cells (Fig. 4h), indicated a potential role of LrNK & LrT cells in the pathogenesis of HBV-associated liver diseases. Through this comprehensive analysis, we demonstrate that CXCR6 marks LrT and LrNK cells, including MAIT, CXCR6^+^ CD8^+^ Trm cells, γδ T cells and CXCR6^+^ TrNK cells, while they display distinct distribution and function in the liver.

### scRNA-seq reveals monocyte cell heterogeneity in human liver

**Fig. 5:**
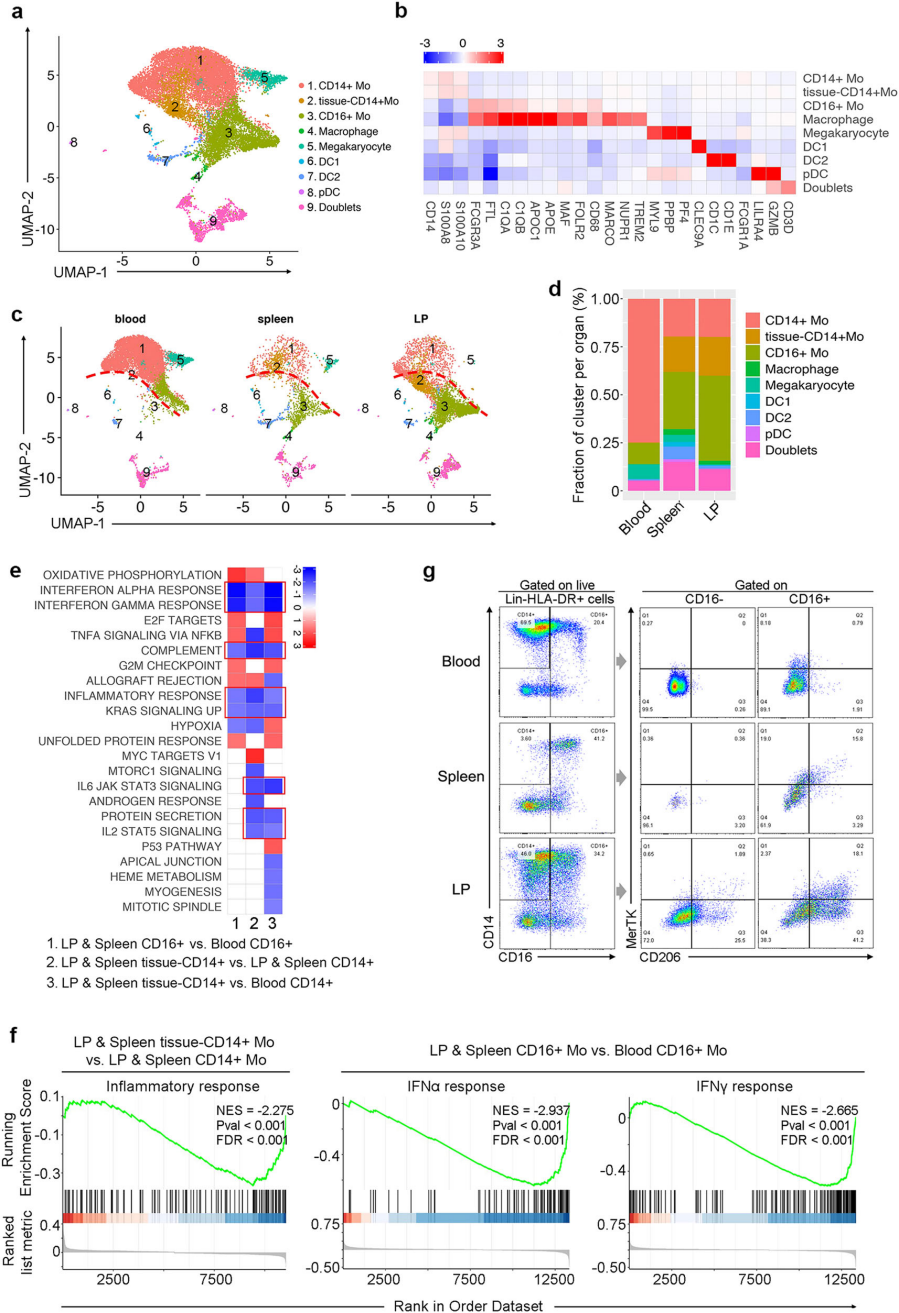
scRNA-seq revealed monophagocytic cell heterogeneity in human liver. **a** UMAP analysis of human myeloid cells showing eight clusters. **b** Heatmap showing significant differentially expressing genes among eight myeloid cell subsets. Selected gene names are labeled at the bottom. **c** UAMP plots of the myeloid cell subsets colored by their tissue source. **d** The proportion of myeloid cell subsets in human blood, spleen and liver perfusion. **e** GSEA analysis of liver-derived CD14^+^ and CD16^+^ monocytes vs. blood monocytes showed enriched pathways from hallmark gene sets. **f** The GSEA plot of inflammatory and IFNα and IFNγ response pathway downregulated in tissue-derived CD14^+^ and CD16^+^ monocyte subsets, respectively. **g** Flow cytometry gating strategy to identify CD14^+^ monocyes, CD16^+^ monocyes and macrophages in human blood, spleen, and liver using markers identified by scRNA-seq data. Data shown are a representative analysis of four healthy liver perfusions.

Eight myeloid cell clusters could be identified and visualized in the UMAP projection (Fig. 5a) basing on typical marker expression (Fig. 5b; Supplementary Table S7). C1 identifies classic CD14^+^ monocytes. C2 identifies a tissue-derived CD14^+^ monocyte subset which was mainly present in the spleen and liver (Fig. 5c). C3 identifies CD16^+^ monocytes, and C4 identifies macrophages. C5 identifies megakaryocytes. C6–C8 represents various DC subsets including cDC1, cDC2, and pDC, respectively. C9 expresses markers of monocytes, T and B cells, suggesting the presence of mixed multiplets.

Tissue-residence patterns for myeloid cell subsets were also examined (Fig. 5c). Megakaryocytes were only found in blood and spleen. Classic CD14^+^ monocytes, which are the major myeloid population in blood, were reduced in spleen and liver. By contrast, the tissue-CD14^+^ monocytes, CD16^+^ monocytes and macrophages were relatively enriched in spleen and liver but were notably absent from blood. DCs represented only a minor cell population, but their proportion in spleen was elevated compared to blood and liver (Fig. 5d). These findings highlight the importance of tissue residence to the distribution of myeloid cell populations.

Environmental cues can finely tune the TrICs, especially the myeloid cell populations. Here, we noticed that for both CD14^+^ and CD16^+^ monocytes, the tissue-derived cells differed with their blood counterpart in the UMAP plot (Fig. 5c), indicating underlying transcriptomic differences (Supplementary Table S8). GSEA of the spleen and liver CD14^+^ and CD16^+^ monocytes as compared with blood CD14^+^ and CD16^+^ monocytes showed a significant common downregulation of interferon (IFN)-α and IFN-γ responses, complement pathway, inflammatory response and IL2-JAK-STAT5, IL6-JAK-STAT3, and P53 pathway (Fig. 5e, f). These findings probably reflect the notion that liver is an immune tolerant organ which would limit the hepatic inflammatory activation.

Finally, through flow cytometry we observed that PB mainly contains CD14^+^ classical monocytes while spleen and LP contain more CD16^+^ monocytes especially for MerTK^+^ and CD206^+^ (*MRC1*) monocyte and macrophage, which matched our scRNA-seq data and previous studies^22^ (Fig. 5g). Overall, this comprehensive analysis confirms that distinct CD14^+^ monocyte, CD16^+^ monocyte, and macrophages are the primary monocyte subsets enriched in the healthy liver, where they adapt to the unique immune tolerant environment.

### Comparative transcriptomic profiling of human B cell and PC subsets

**Fig. 6:**
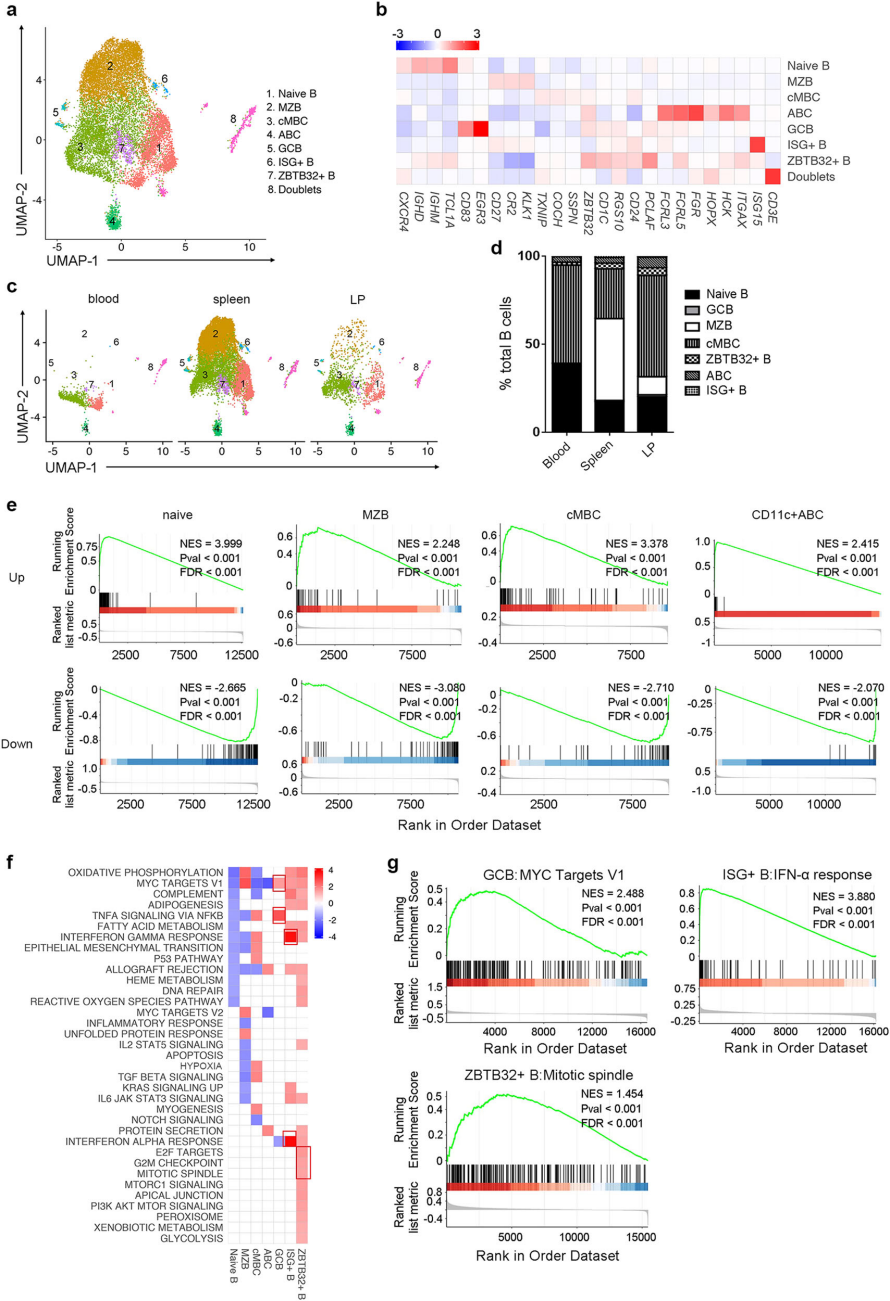
scRNA-seq revealed B-cell heterogeneity in human spleen and liver. **a** UMAP analysis of human B cells showing seven clusters. **b** Heatmap showing significant differentially expressing genes among seven of the B subsets. Selected gene names are labeled at the bottom. **c** A UMAP plot of B-cell subsets by their tissue source. **d** The proportion of Bcell subsets in human blood, spleen, and liver perfusion (LP). **e** GSEA analysis of scRNA-seq defined B-cell clusters vs. previous reported Naive B, MZB, cMBC, and ABC signatures. Naive B, MZB, and cMBC signatures were generated with GSE64028, ABC signature was generated with GSE110999. **f** GESA analysis of GCB, ISG^+^ B, and ZBTB32^+^ B-cell subset showed enriched pathways from hallmark gene sets. **g** The GSEA plot of MYC targets, IFNα, and mitotic spindle pathway enriched in GCB, ISG^+^ B, and ZBTB32^+^ B cells, respectively.

Clustering analysis identified 7 of B-cell clusters visualized using UMAP (Fig. 6a) basing on typical marker expression (Fig. 6b, Supplementary Table S9). C1 was naïve B-cell subset. C2 identified marginal zone B (MZB) cells enriched in the spleen. C3 expressed *TXNIP*, *COCH*, and *SSPN* and identified the classical memory B (cMBC) cells. C4 expressed *ITGAX* and identified age-associated B cells (ABCs)^23^. C5 identifies germinal center B (GCB) cells. C6 highly expressing interferon stimulated genes (ISG) and was named ISG^+^ B cells. C7 highly expressed ZBTB32 and was named ZBTB32^+^ B cells.

Tissue-residence patterns of B-cell subsets were assessed through UMAP comparison (Fig. 6c, d). Spleen clearly contains the largest number of B cells. Naïve B cells comprised a large proportion of the B-cell compartment in all tissues. The MZB subset was absent from blood and represented a major B-cell subset in spleen. MZB cells were also present in the LP. The proportion of cMBC was similar in blood and LP, but reduced in spleen. ABC was a distinct subset present in blood, spleen, and LP. GCB, ISG^+^ B, and ZBTB32^+^ B cells are rare populations mostly enriched in spleen and liver, except for small number of ZBTB32^+^ B cells in blood (Fig. 6c, d). We also noticed that both cMBC (C3) and ZBTB32^+^ B cells (C7) in blood differed from their tissue counterparts in the UMAP plot (Fig. 6c), indicating possible transcriptomic differences (Supplementary Table S9).

There is no agreement on specific markers of human B-cell subsets. To confirm the identification of major B-cell populations, we extracted gene expression signatures from previous reports on naive, MZB, cMBC, and ABC^24,25^, and examined the selective enrichment of these signatures in scRNA-seq defined B-cell subsets. Indeed, the GSEA analysis showed that both upregulated and downregulated gene sets matches well in naive, MZB, cMBC, and ABC cells, indicating that our clustering faithfully revealed the heterogeneity of human B-cell populations (Fig. 6e).

GSEA of the GCB, ISG^+^ B, and ZBTB32^+^ B cells compared to other B-cell subsets was also conducted to probe their unique functions (Fig. 6f). GSEA of GCB showed enrichment for hallmark gene sets, including Myc targeted pathway and TNF signaling via NFkB, apoptosis and inflammatory response. Indeed, we confirmed increased expression of Myc-targeted genes from GCB cells (Fig. 6g). These genes and pathways are features of GCB from the light zone^26^. GSEA of ISG^+^ B showed enrichment of IFNα and IFNγ responses (Fig. 6f, g). GSEA of ZBTB32^+^ B showed enrichment of pathways, including mitotic spindle, G2M checkpoint, and E2F targets (Fig. 6f, g), indicating a population of proliferative B cells.

**Fig. 7:**
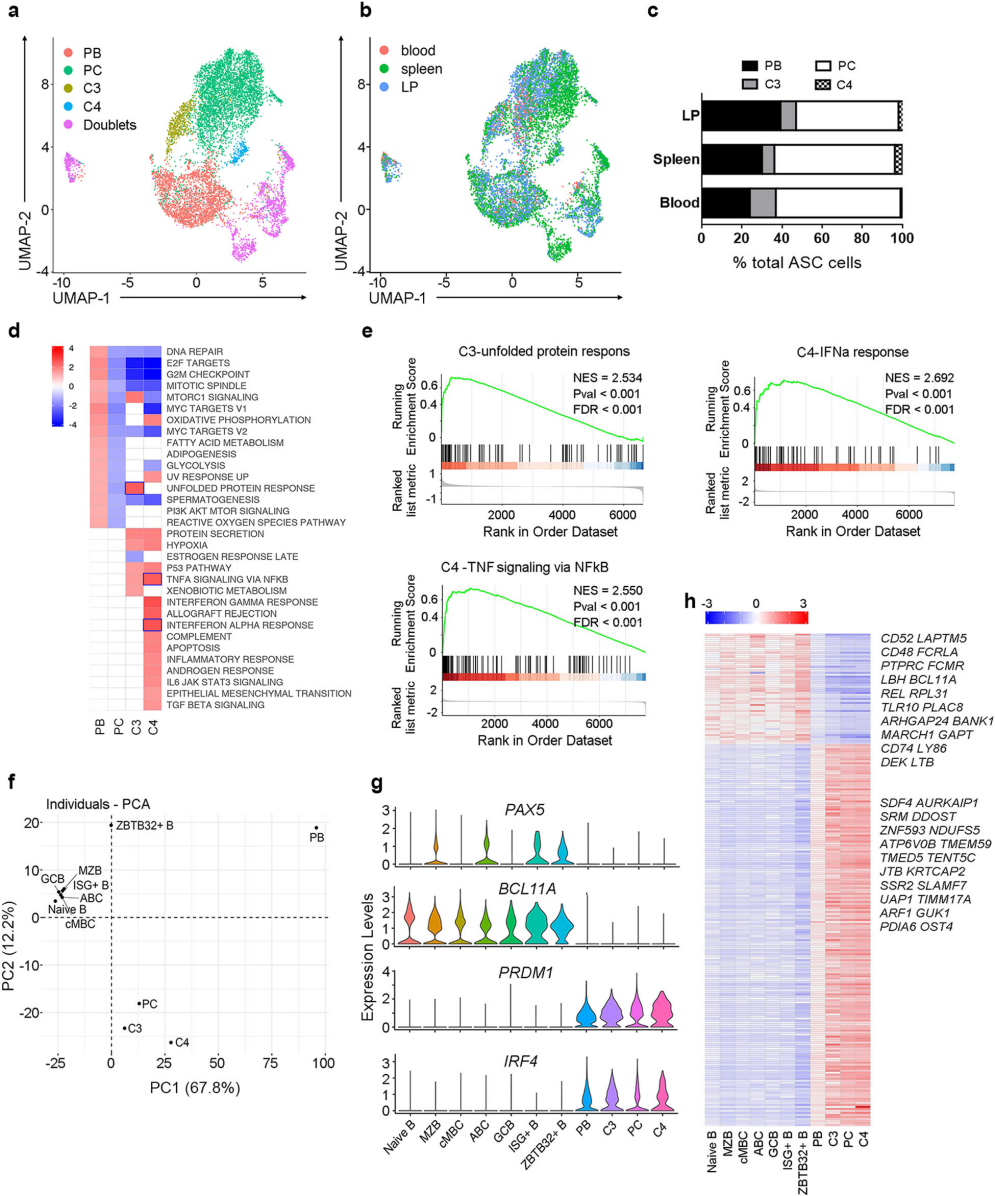
Differential transcriptome profiling of human B cell and ASC subsets at single-cell resolution. **a** UMAP analysis showing four clusters of ASCs. **b** UMAP plots of ASCs cell subsets colored by their tissue source. **c** The proportion of ASCs subsets in human blood, spleen, and liver perfusion. **d** GESA analysis of ASCs subset showed enriched pathways from hallmark gene sets. **e** The GSEA plot of unfold protein response, IFNα response, and TNF signaling via NFκB pathway enriched in PC-C3 and PC-C4 cells. **f** PCA analysis for human B cell and ASC subsets based on mean expression of variably expressed genes. **g** Violin plots showing the expression of selected transcriptional factors such as *PAX5*, *BCL11A*, *PRDM1*, and *IRF4* between B cell and ASC subsets. **h** Heatmap showing significant differentially expressing genes among seven B cell subsets and four ASC subsets. The gene names in the top 20 most DEGs are labeled on the right.

Four of PC, the antibody-secreting cell (ASC), clusters could be identified and visualized in UAMP as follows (Fig. 7a) basing on typical gene expression (Fig. 7a; Supplementary Fig. S10a, Table S10): C1 was identified as plasmablasts (PBs) and C2 as PCs. C3 and C4 were PC clusters with unique transcriptomic features. GSEA of C3 showed enriched pathway for unfolded protein response and protein secretion (Fig. 7b, c), indicating an active antibody producing population, while GSEA of C4 showed enriched pathway for IFNα response and inflammatory response, indicating a cytokine-stimulated population in vivo (Fig. 7b, c). The distributions of ASC clusters across blood, spleen, and LP were similar with approximately one-third identified as cycling plasmablasts (C1), however, the immune activated C4 resided only in the tissue and not blood (Fig. 7d, e).

We then compared B and ASCs transcriptomes so as to search key genes determining ASC differentiation. ASCs are in an active metabolic state reflected by their higher UMI and gene counts (Supplementary Fig. S10b). Almost 20% in PBs and 50% in PCs of the gene contents are immunoglobulin genes (Supplementary Fig. S10c), indicating that they are dedicated antibody producers. After exclusion of immunoglobulin genes, PCA analysis identified four ASC clusters distant from the B cell clusters, indicating that ASCs hold unique transcriptional signatures in addition to their higher levels of immunoglobulin gene expression (Fig. 7f). *BCL11A* and *PAX5* are expressed by B cells but lacking in ASCs. By contrast, *PRDM1* and *IRF4* are preferentially expressed by ASCs compared to B cells (Fig. 7g). Further analysis of ASC- and B cell-associated DEGs identified 190 genes enriched in ASCs and 55 genes enriched in B cells, consistent with their unique immune functions (Supplementary Table S11). The list shows specific markers such as MHC class II molecules, *MS4A1*, *CD79B* for B cells, and *TNFRSF17*, *CD38*, *XBP1*, etc. for ASCs. The ASC gene signatures indicated their activities on antibody production, since many highly expressed genes in ASCs are related to protein translation, modification and trafficking (Fig. 7h). Taken together, we gave a global glimpse on B cell and ASC subsets in various human tissues.

## Discussion

Our study provides the first transcriptional atlas of LrICs and identifies their heterogeneity in healthy donors using paired spleen and blood as reference tissues, which enabled an analysis of subset structure and corresponding specific genes, distribution, and immune functions of various cell subsets in these organs (Table 1). Compared to previous studies^5,12,13,27^, our data extend significantly the identity and liver residency of human immune cell subsets. Our findings will be a rich source for evaluation of transcriptional profiles in various tissue immune cells and diseases, and provide comparative scRNA data for future studies.

**Table 1:** Summary of the critical features of each cell type identified in the study.

Category	Subcategory	Counts			Markers	Tissue distribution
		Blood	Spleen	LP		
CD4 T	Naive/CM	1886	1026	801	*SELL, CCR7, SOCS3, MYC, LEF1, TSHZ2, MAL, NOSIP, TCF7*	Blood, spleen, liver
	Tem	1719	1630	1247	*LTB, IL7R, CD52, TNFRSF4, AQP3, TIMP1*	Blood, spleen, liver
	T_FH_	57	1110	244	*GPR183, LTB, CD40LG, CXCR5, MALAT1*	Spleen, liver
	Treg	289	278	177	*FOXP3, RTKN2, CTLA4, FANK1, IL2RA, ICA1, ARID5B, CORO1B, GBP2*	Blood, spleen, liver
CD8 T	Naive/CM	597	350	234	*SELL, CCR7, MYC, LEF1, TSHZ2, MAL, NOSIP, TCF7, RCAN3,SOCS3*	Blood, spleen, liver
	Tem	448	490	387	*CD8A/CD8B, CXCR3, CD52, ITGA1*	Blood, spleen, liver
	Trm	8	976	3010	*KLRG1, CXCR6, KLRD1, CCL4, CCL5, XCL2, GZMK, CMC1*	Liver, spleen
	T_FC_	5	1164	241	*CXCR5, CD8A, CD8B*	Spleen, liver
	MAIT	5	1114	3027	*KLRB1, KLRG1, NCR3, SLC4A10, CXCR6, JAML, IL7R, CD160*	Liver, spleen
	Tc	194	215	563	*CX3CR1, LGALS1, FGFBP2, GNLY, GZMB, PTGDS, SPON2, PRF1, ZNF683, NKG7*	Blood, spleen, liver
γδ T		0	266	92	*CD8A/B-, TRDC/TRGC2*+*,CMC1, ITGAD*	Liver, spleen
ILC	TrNK	5	174	1149	*XCL1, XCL2, CCL3, KLRC1, CD160, IL2RB, GSTP1, FCER1G, TYROBP, CXCR6, GZMK*	Liver, spleen
	cNK	278	1447	3137	*CX3CR1, LGALS1, FGFBP2, GNLY, GZMB, PTGDS, SPON2, PRF1, TYROBP, NKG7, FCGR3A, PRF1*	Blood, spleen, liver
	ILC	0	84	17	*LST1, FXYD5, OTUD5, SPINK2, IL1R1, ALDOC, IL4I1, TNFRSF4, IL7R, TNFSF13B*	Spleen, liver
Cycling NK & T		119	691	2721	*STMN1, HIST1H4C, HMGB2, TUBA1B, TUBB, TYMS, PCLAF, PCNA, MKI67, RRM2, DUT*	Liver, spleen
Myeloid cells	CD14+ Mo	6772	440	1100	*LGALS2, VIM, CLEC4E, CRIP1, S100A8, S100A9, S100A12, RNASE2, PLBD1, CLU, RETN, VCAN, MNDA, LYZ*	Blood, spleen, liver
	tissue-CD14+ Mo	24	414	1124	*MT1X, MT1E, MT1G, VCAN, FOLR3, IER2*	Liver, spleen
	CD16+ Mo	1005	665	2460	*HLA-DPB1, HLA-DPA1, CD74, HLA-DRB1, MS4A7, C1QA, C1QB, C1QC, FCGR3A, RHOC, SMIM25, CSF1R*	Blood, spleen, liver
	Macrophage	1	66	111	*FTL, SELENOP, HMOX1, FABP5, CD5L, APOE, APOC1, FABP4, NUPR1, MARCO, LGMN, SLC40A1, MRC1*	Liver, spleen
	Megakaryocyte	692	83	9	*PPBP, PF4, GNG11, CAVIN2, NRGN, MYL9, SPARC, TUBB1, ITGA2B, PIG6B*	Blood, spleen
	cDC1	9	55	27	*CLEC9A, PTTG1, HIST1H4C*	Spleen, liver
	cDC2	68	148	87	*HLA-DQA1, HLA-DQB1, CD1C, PPA1, FCER1A*	Blood, spleen, liver
	pDC	5	29	7	*LILRA4, TCF4, CCDC50, IRF8, BCL11A, ITM2C, C12orf75, IRF7, SPIB*	Spleen, liver
B cells	Naïve B	401	1921	364	*TCL1A, IGHD, IGHM, FCER2, CXCR4, APLP2, PLPP5, IL4R*	Blood, spleen, liver
	MZB	4	5156	184	*SMIM14, CD27, CR2, KLK1, ITM2C, DNASE1L3, HES4, ABI3, CHI3L2*	Spleen
	cMBC	576	3125	1036	*TSC22D3, CRIP1, COCH, CD27, ITGB1, ANXA2, IGHA2*	Blood, spleen, liver
	CD11c+ ABC	33	353	107	*FGR, FCRL5, MPP6, FCRL3, MACROD2, HSPB1, CELC2D, CD72*	Spleen, liver
	GCB	0	96	22	*EGR3, CD83, DUSP2, EGR2, NR4A1, MYC, TNF, NFKBID, NFKBIA, CD69*	Spleen, liver
	ISG+ B	1	70	7	*ISG15, IFI6, IFIT3, IFIT1, IFI44L, MX1, ISG20, IRF7, SAMD9L, IFI44*	Spleen, liver
	ZBTB32+ B	17	356	79	*ARPC1B, CD1C, PCLAF, ZBTB32, STAC3, DUS2, PTPN1, CPNE5*	Spleen, liver
Plasma cells	PB	197	1337	963	*UBE2C, TOP2A, CKS2, KPNA2, CDC20, UBE2S, TUBB4B, PTTG1, HIST1H4C, RRM2, PCLAF, DUT, MKI67*	Spleen, liver
	PC	510	2669	1254	*JCHAIN, DPEP1, IGHA2, IGKC*	Blood, spleen, liver
	C3-PC	106	271	191	*HERPUD1, SLC3A2, PSAT1, SLC1A5, EIF1, MTHFD2, WARS, EIF4EBP1, HSPA5*	Blood, spleen, liver
	C4-PC	8	171	48	*IGLL5, IGLL1, SSR4, SDC1*	Spleen, liver
**Total**		**16,** **039**	**28,** **440**	**26,** **227**		

Our data give novel clues for categorization of both T and NK cell subsets based on unique expression patterns of several chemokine receptors. For CD8^+^ T cells, CCR7 defines Tnaive and Tcm cells, CXCR3 does for circulating Tem cells, CXCR6 does for MAIT, Trm, and γδ T cells and CX3CR1 does for GZMB^+^ Tc cells. This new classification shows several advantages over previous T cell category basing on CD45RA and CCR7^28^. First, our categorization fits accurately with the current understanding of immune surveillance because our data covers PB, secondary lymphoid tissue (spleen), and peripheral tissue (liver). By contrast, the CD45RA and CCR7 based T cell categorization was mainly derived from PB and did not describe organ-derived memory T cells. Second, our T cell categories partly overcome Tem heterogeneity by more clear definition of MAIT cells, CXCR3^+^ Tem cells, CXCR6^+^ Trm cells and CX3CR1^+^ Tc, which share the CD45RA^−^CCR7^−^ phenotype based on previous classification^29^. Third, CXCR6 is likely a better marker to define Trm than CD69 (and/or CD103) which, as tissue residence marker, is well-known to be an activation marker and insufficiently defines Trm^30^. This notion has been supported by several previous studies^31–33^. Fourth, CX3CR1 is proposed as a useful marker to define CD4^+^ and CD8^+^ Tc cells in the study because it is co-expressed with functional cytotoxic granules such as *GZMB, GNLY, PERF*, etc. and is recently used to define cytotoxic subsets^34^. Finally, CXCR6 and CX3CR1 define LrNK and cNK cells in similar manner, which is consistent with distinct expression of TFs such as *EMOES*, *PLZF*, and *TBX21*, and granules such as *GZMK* and *GZMB*, respectively, in agreement with early reports characterizing LrNK cells^6^. Thus, we identify CXCR6 as liver-resident marker for both T and NK cells, and characterize human liver-resident NK & T cells at the single cell level for the first time. We also dissect their immune functions through addressing the core hallmark genes expressed by liver-resident T & NK cells, including adhesion and integrin markers, chemokine and their receptors, co-inhibitory markers, killer lectin-like receptors, and transcriptional factors *EOMES*, *ZBTB16*, *IKZF2*, and *RORC*. These CXCR6^+^ T and NK cells also show a self-renewal proliferative capacity, and play key roles in local immune and inflammatory response as well as chemotaxis mediation. Overall, the present category of T and NK cells based on chemokine receptors unifies the liver-resident T and NK cell biology.

Three functionally distinct monocyte subsets have been characterized in human PB, including classical CD14^high^CD16^−^ monocytes, CD14^high^CD16^+^ intermediate monocytes, and CD14^low^CD16^+^ nonclassical monocytes^35^. Our data revealed the CD14^+^ classical monocyte, tissue-CD14^+^ monocytes, CD16^+^ monocyte subsets and macrophage in human liver and spleen, and provided a map of the baseline hepatic monocyte framework. Different from CD14^+^ classical monocytes which are the major component in PB, the proportion of tissue-CD14^+^ monocytes and CD16^+^ monocytes is much greater in the liver and spleen than in blood. Notably, our monocyte categorization is not identical to previous monocyte subsets in human blood^17^ and pro-inflammatory macrophages observed in mouse and human liver^8,12^. In addition, our results revealed several downregulated pathways specifically associated with liver-derived monocytesas compared to PB CD14^+^ and CD16^+^ monocytes such as inflammatory response and IFN-α and IFN-γ responses. These findings likely reflect the notion that liver is an immunotolerant organ which limit the hepatic inflammatory activation. The validation of these pathways greatly contributes to better resolution of hepatic monocytes and macrophages. Taken together, our data characterized the differences and relations between hepatic monocytes and macrophages, and could be used as reference data for future comparison studies on their physiological and pathological significances.

Our analysis also revealed the heightened expression of MTs and chemokines in liver-derived immune cell subsets. MTs have emerged as an important, yet largely underappreciated component of the immune system to regulate the activation, proliferation and differentiation potential of immune cells through regulating the homeostasis of zinc and copper^36^. Exposure of MTs impacts the ability of CD8^+^ T cells to proliferate and mount cytotoxic responses against allogeneic target cells^37^. Combination with these studies, our data highlight several aspects: (1) MTs enrichment in hepatic immune cells is possibly due to the Zn-rich environment in the liver; (2) higher expression of MTs in hepatic immune cells may modulate hepatic immune defenses and tolerance; and (3) MTs may be required to sequester Zn in the liver in case of infection. Addressing these questions will be helpful for understanding the functional roles of MTs in immunological fitness of human liver.

Finally, we produced the first scRNA atlas of large numbers of human B cells and ASCs and confirmed the existence of previously reported naive B, cMBC, CD11c^+^ ABC, and MZB subset and PB and PC, and identified three additional B-cell subsets including GCB cells, cycling ZBTB32^+^ memory B cells and ISG^+^ B cells as well as two populations of previously unidentified PCs. This comprehensive dataset of human B cells and ASCs enables the comparison of subset transcriptomes and identify novel subset specific genes which enable better discerning the B cell and ASC subsets. These newly identified signature genes would not only be helpful for future characterization of B-cell subsets, but also provide insights for their functions. This study, therefore, gives a global glimpse on B cells in human tissues, supplementing the previous data on mice B cells and ASCs^38^.

This study is limited by several aspects. First, although LP from transplanted livers contains available liver-resident or enriched cells^39,40^, it may not exactly reflect hepatic immune cells, which possibly causes the difference of cell identity including macrophages. Second, many genes were found to be uniquely expressed in various cell subsets, but their functions remained unclear. Third, more comprehensive bioinformatics analysis is required to reveal more tissue specific immune characteristics. Nonetheless, our transcriptional atlas of LrICs at the single-cell level provides a framework for understanding the basis of liver immune responses and serves as a powerful resource for the development of immune cell-based diagnostics and therapeutic methods especially for liver diseases. The use of paired LP, spleen and blood adds additional dimensions supporting the determination of novel immune subsets and markers, comparison of potential inter- and intra-organ immune functional variations, identification of differentiation pathways, and other uses.

## Materials and methods

### Human tissue cell isolation

For the scRNA-seq characterization, human tissue samples including LP, spleen, and PB used in this study were obtained from three adult donors with liver transplantation with approval from the Institutional Review Board and the Ethics Committee of Shenzhen Third People’s Hospital, Guangdong province and Beijing 302 Hospital, Beijing, China. The written informed consent was obtained from each donor.

Immune cells from human samples were isolated according to previous protocols^41^. Complete RPMI media were used for all cell isolations. In brief, PBMCs (*n* = 26) were isolated by ficoll-hypaque density gradient centrifugation from heparinized blood of enrolled subjects. Spleen (*n* = 15) was first ground on ice and the cells were collected and filtered. LP was directly filtered and concentrated by centrifugation (750*g*, 15 min, 20 °C), and then layered onto Ficoll.

In scRNA-seq assay, human CD45^+^ cells from LP, spleen cells, and PBMCs were isolated with the anti-CD45 magnetic-activated cell sorting kit, and cells were counted three times by three individuals independently and a mean of 22, 000-24, 000 live cells were finally used for 10× Genomics. The remaining cells were cryopreserved in 90% fetal calf serum (FCS) plus 10% DMSO for use in subsequent assays. For additional validation assays, liver biopsies were also collected from 12 chronic hepatitis B (CHB) and 10 HBV-associated liver cirrhosis (LC) patients, and 8 healthy liver tissue samples were obtained from donors whose livers were used for transplantation. In addition to the tissues used for pathological evaluation, these liver biopsy specimens were homogenized for the validation assay of T cells, NK cells, and monocytes using flow cytometry^41^.

### RNA-Seq library construction using the 10× genomics platform

The scRNA-Seq libraries were prepared with Single Cell 3′ Reagent Kit v2 (10× Genomics; 120,237, 120,236, and 120,262) following the user guide provided. Briefly, 22, 000 cells were encapsulated into droplets at a concentration of 2, 000 cells/μl. After the encapsulated cells were lysed and reverse transcripts (53 °C for 45 min; 85 °C for 5 min) were performed in a 96-Well Thermal cycler (Thermo Fisher Scientific; 4375786), droplets were broken and barcoded-cDNA was purified with DynaBeads (Thermo Fisher Scientific; 37002D), followed by 14-cycles of PCR-amplification (98 °C for 3 min; [98 °C for 15 s, 67 °C for 20 s, 72 °C for 1 min] × 14 cycles; 72 °C for 1 min). The amplified-cDNA was then fragmented, ligated with adapter and sample index, and selected with SPRI beads (Beckman Coulter; B23318) to average 300 bp size. The constructed library was sequenced on Illumina Hiseq XTEN platform.

### Alignment, UMI counting, and sample aggregating

The Cell Ranger Software Suite (Version 3.0.2) was used to perform sample de-multiplexing, barcode processing, and single-cell 3′ UMI counting with human GRCh38 as the reference genome. Specifically, splicing-aware aligner STAR^42^ was used in FASTQs alignment. Cell barcodes were then determined based on distribution of UMI count automatically. Finally, gene-barcode matrix of all three donors was integrated with Seurat v3^43^ to remove batch effect across different tissue and donor. Following criteria were then applied to each cell, i.e., gene number between 200 and 6000, UMI count above 1000 and mitochondrial gene percentage below 0.1. After filtering, a total of 77, 099 cells (475/8267/5646 cells for blood/spleen/LP of donor 1; 7562/9609/11,488 cells for blood/spleen/LP of donor 2; 9239/13,958/10,855 cells for blood/spleen/LP of donor 3) were left for following analysis. In the following analysis, a total of 6393 cells were further excluded due to doublets, the remaining 70,706 cells were finally entered into the analysis of clusters.

### Dimensionality reduction and clustering

The filtered gene-barcode matrix was analyzed by PCA. Then Uniform Manifold Approximation and Projection (UMAP) was performed on the top 50 principal components for visualizing the cells. Meanwhile, graph-based clustering was performed on the PCA-reduced data for clustering analysis with Seurat v3. For different cell types, cells were grouped based on known markers and analyzed with Seurat v3 in the similar manner.

### Differential analysis for clusters

MAST^44^ in Seurat v3 was used to perform differential analysis. For each cluster, DEGs were generated relative to all of the other cells.

### Identification of cluster-specific genes and marker-based classification

To identify genes that are enriched in a specific cluster, the mean expression of each gene was calculated across all cells in the cluster. Then each gene from the cluster was compared to the mean expression of the same gene from cells in all other clusters. Genes were ranked based on their expression difference, and the top DEGs from each cluster were examined. For hierarchical clustering, pair-wise correlation between each cluster was calculated, and centered expression of each gene was used for visualization by heatmap. Classification of immune cell subsets was inferred from the annotation of cluster-specific genes. The identification markers for each subset are summarized in Supplementary Tables S3–S9.

### Gene functional annotation

For DEGs, Gene ontology (GO), KEGG pathway analyses, and Gene Set Enrichment Analysis (GSEA)^45^ were performed with clusterProfiler^46^, which supports statistical analysis and visualization of functional profiles for genes and gene clusters. In GSEA analysis, 50 hallmark gene sets in MSigDB^47^ were used for annotation. To verify the validity of clustering result for B cells, GSEA was used to discover the correlation of marginal zone B (MZB) cells, classical memory B cells (cMBC), Naïve B and CD11c^+^ ABCs with signatures of these cell types calculated from published dataset (GSE64028 for MZB, Naïve and classical memory B cells, GSE110999 for CD11c^+^ ABC cells), respectively^24,25^.

### Single-cell signatures

Tissue-resident NK & T core signatures score was calculated for all NK & T cells and visualized with Single-Cell Signature Explorer^20^. Since Single-Cell Signature Scorer only consider positive marker for signature calculation, we referred to the method^48^ which consider both upregulated and downregulated markers.

### Real-time PCR

Lymphocytes total RNA was extracted with TRIzol reagents (Invitrogen) in accordance with the manufacturer’s instructions, and further reverse-transcribed into cDNA with a High-Capacity cDNA Reverse Transcription Kit (Takara). The expression levels of MT2A were determined by qPCR analysis using Power SYBR Green PCR Master Mix (Takara). The real-time PCR primers set for MT2A detection were as follows: forward (5′-GAGTGCAAATGCACTTCGTGCAA-3′) and reverse (5′-GCGTTCTTTACATCTGGGAGCG-3′), and the data was normalized to actin RNA (5′-CACCATTGGCAATGAGCGGTTC-3′ and 5′-AGGTCTTTGCGGATGTCCACGT-3′).

### Flow cytometry

The fluorochrome conjugated antibodies or regents from Biolegend, BD Bioscience, eBioscience and R&D Systems were used in the study. For human PBMCs and various tissue derived lymphocytes, dead cells were excluded using fixable viability dye eFluor 450 (eBioscience). The left live CD45^+^ cells were analyzed for phenotypic expression by BD LSRFortessa, and the data were further analyzed with FlowJo software (TreeStar, San Carlos, CA). For surface marker staining, the cells were incubated with antibodies on ice for 30 min and then washed and fixed with further analysis. For the staining of transcriptional factors, the cells were stained with surface marker first and then permeabilized using a Cytofix/Cytoperm kit (BD Bioscience) and stained for intracellular protein. For intracellular cytokine detection, freshly isolated cells were incubated with various stimulators followed by Golgi-stop for additional 6 h. The cells were then collected for surface marker staining, followed by cell permeabilization and intracellular cytokine staining. For CD107a staining, the cells were incubated with anti-CD107a antibodies since the onset of stimulation. Then the cells were further incubated with BFA for additional 6 h.

### Cell culture and stimulation

Human lymphocytes (1 × 10^6^) were plated in 96-well plates and were cultured in RPMI 1640 complete medium containing 10% FCS (Invitrogen), 2 mM l-glutamine (Invitrogen), 100 U/ml penicillin, 100 μg/ml streptomycin (Invitrogen), and recombinant human 10 ng/ml IL-2 (PeproTech, Rocky Hill, NJ). For T and NK cell stimulation, IL-12/IL-18 (50 ng/ml; PeproTech) was supplemented into the medium for 6 h, followed by intermediate FACS analysis to detect cell activation. For measuring the cytokine secretion, the cultured cells were collected and analyzed by FACS after addition of Golgi stop solution (BD Bioscience) for at least 6 h.

### Statistical analysis

Data were analyzed using GraphPad Prism software version 5.0 (GraphPad software, San Diego, CA, USA). Data represent mean ± SEM. Results were considered significant when *P* < 0.05. Multiple comparisons were first made among the three groups using the Kruskal–Wallis *H* nonparametric test. Then the comparisons between two groups were performed using the Mann–Whitney *U* test. The comparisons in the same individual were performed with Wilcoxon’s matched-pairs test.

## Supplementary information

Supplementary Information

Supplementary Tables S3-S11

## Data Availability

All relevant data are available from the authors. Single-cell RNA-seq data have been deposited in the Short Read Archive under accession number GSE125188.

## Supplementary information

**Supplementary Information** accompanies the paper at (10.1038/s41421-020-0157-z).
