# Supplementary material for: Single-cell RNA sequencing reveals the heterogeneity of liver-resident immune cells in human
Source: Cell Discov. 2020 Apr 28;6:22. doi: 10.1038/s41421-020-0157-z (PMC7186229; doi:10.1038/s41421-020-0157-z)
Supplement: Supplementary file 1 — Supplementary Information [file 41421_2020_157_MOESM1_ESM.pdf]

Single-cell RNA sequencing reveals the heterogeneity of liver-resident immune cells in human

Running title: the heterogeneity of human liver-resident immune cells

Supplemental Information

A total of 10 of supplemental figures and 11 of supplemental tables are listed in the supplemental information section. Fig. S1 – Fig. S10 and their legends are listed as followings. The Table S1 and Table S2 are also listed in the section, while Table S3-Table S11 showing the DEGs lists of each cell subsets are listed in other excel files.

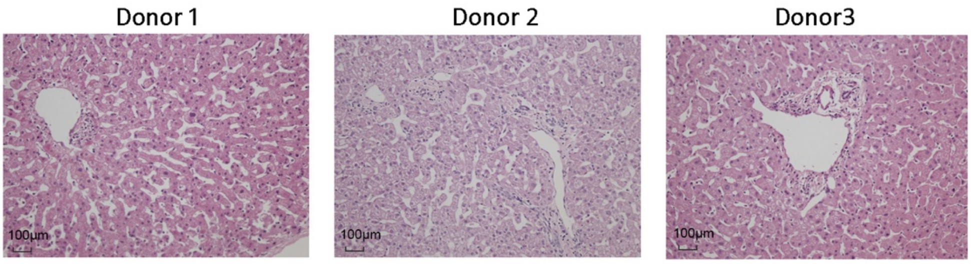

Fig. S1 The histology of the donated liver used in the scRNA-seq study.

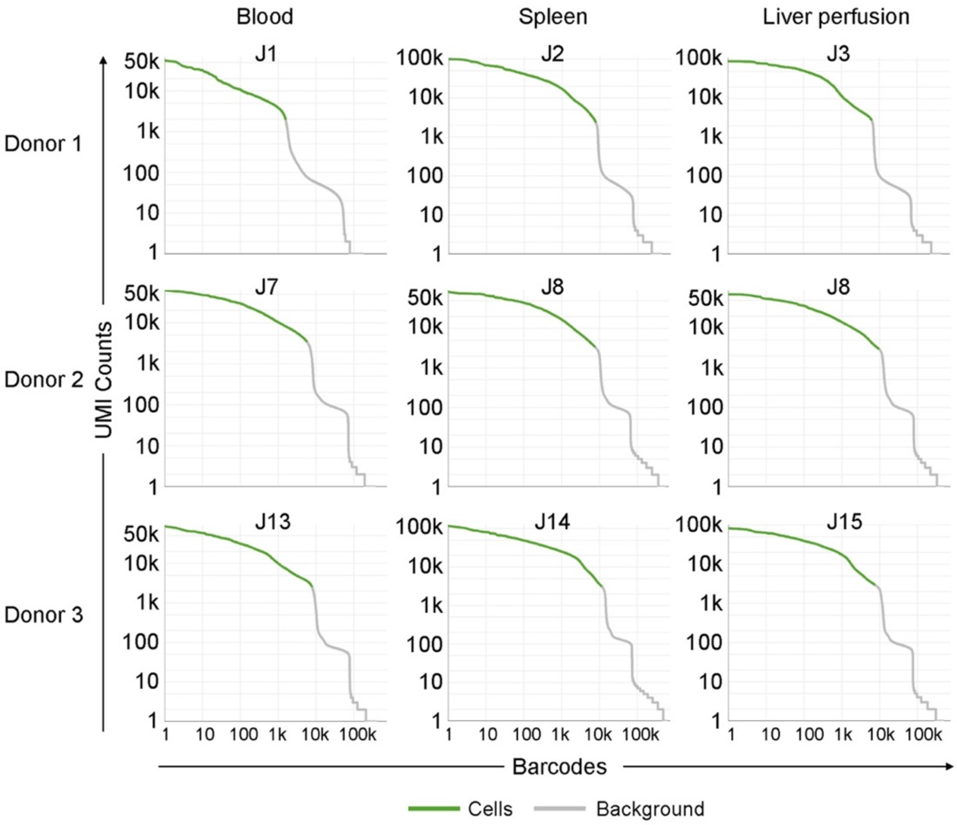

Fig. S2 Summary of estimated number of cells in the scRNA-seq study.

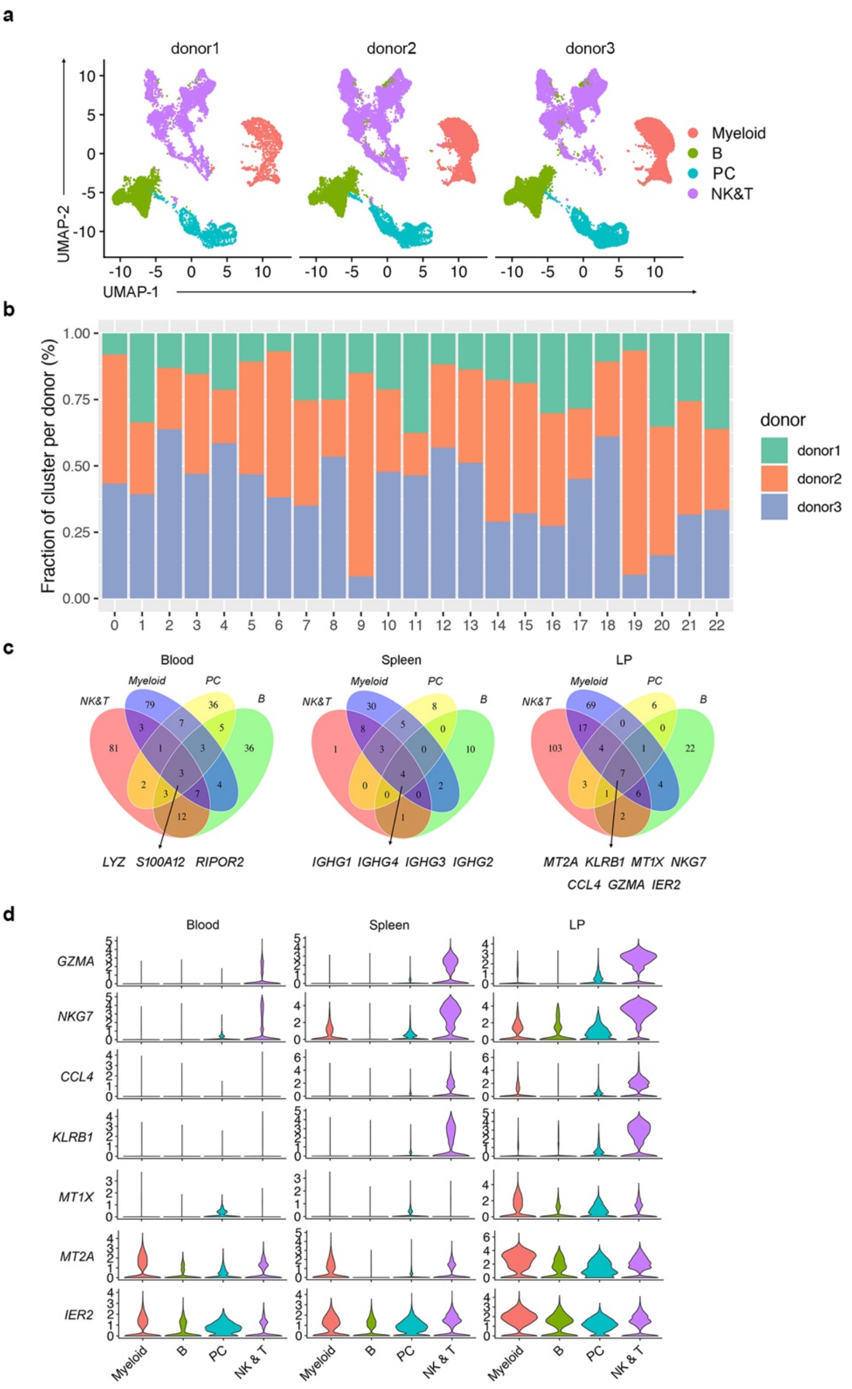

Fig. S3 The tissue distribution of immune cells in human liver perfusion (LP), spleen and blood. (a) A UMAP plot of the immune cells colored by their donor source. (b) Donor distribution of by clusters. (c) Venn plot indicating the distribution of specifically expressed gene counts in various tissues from each major lymphocyte subset including NK&T, B, ASC and myeloid cells. (d) The violin plots showing the expression of *GZMA, NKG7, CCL4, KLRB1, MT1X, MT2A* and *IER2* in each major immune subsets from blood, spleen and LP.

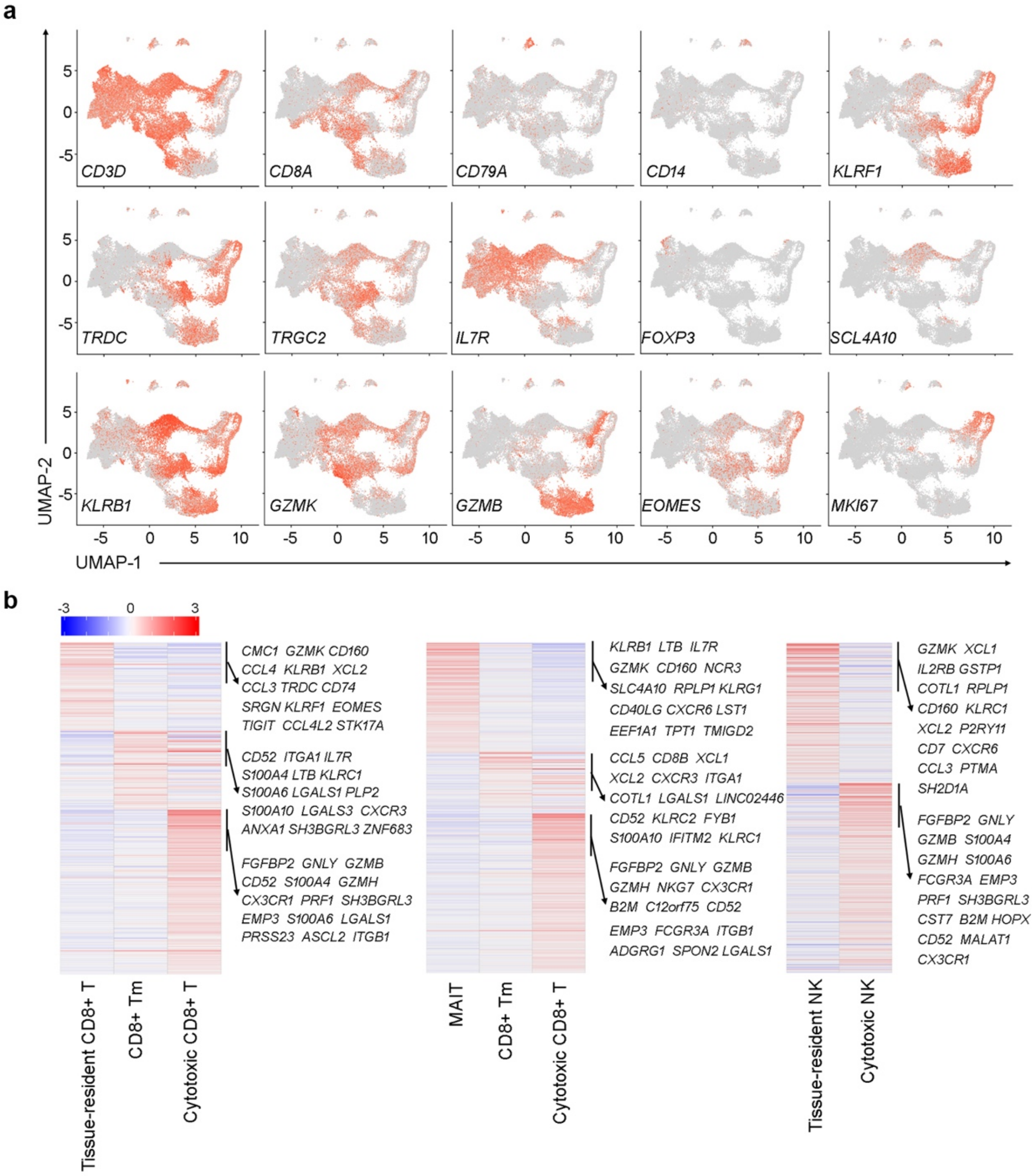

Fig. S4 Analysis of unique markers of NK&T subsets. (a) UMAP plot showing the expression of key markers in various T and NK cell subsets. (b) Heatmaps showing DEGs by various tissue-resident CD8$^+$ T cells and NK cells versus other T cell and NK cell subsets. The top 15 of gene names with *P* values were listed on the right for each subset.

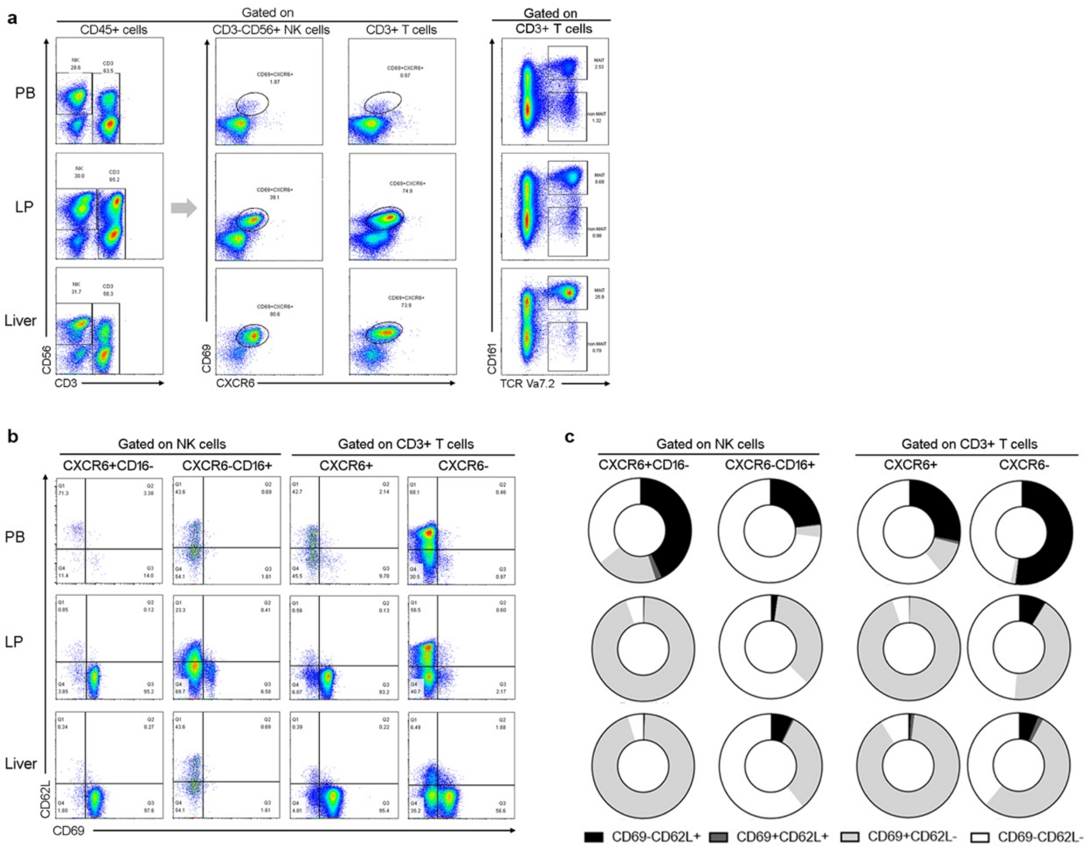

Fig. S5 Characterization of tissue-resident T and NK cells in various tissue. (a) Representative dot plots identified the co-expression of CD69 and CXCR6, and also depicted the proportion of CXCR6$^+$CD69$^+$ tissue-resident T and NK cells and MAIT cells within CD3$^-$CD56$^+$ NK cells and CD3$^+$ T cells, respectively. (b) Representative dot plots showing the expression of CD69 and CD62L on various T and NK subsets. (c) Summary data indicated the proportion of CD62L$^+$ and CD69$^+$ cells within CXCR6$^+$CD16$^-$ LrNK cells, CXCR6$^-$CD16$^+$ cNK cells, CXCR6$^+$ LrT cells and CXCR6$^-$ non-LrT cells from peripheral blood (n = 13), liver perfusion (n = 5) and liver (n = 9), respectively.

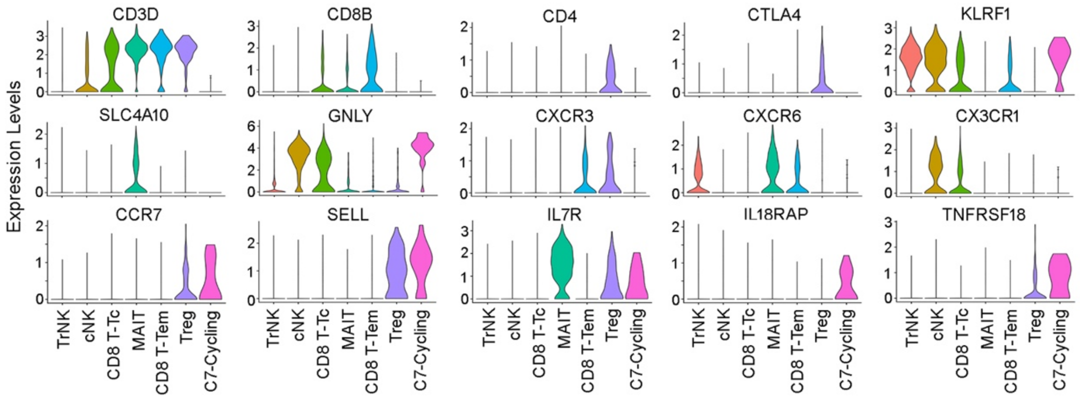

Fig. S6 Violin plots showing the expression of selected markers in various cycling T and NK cell subsets.

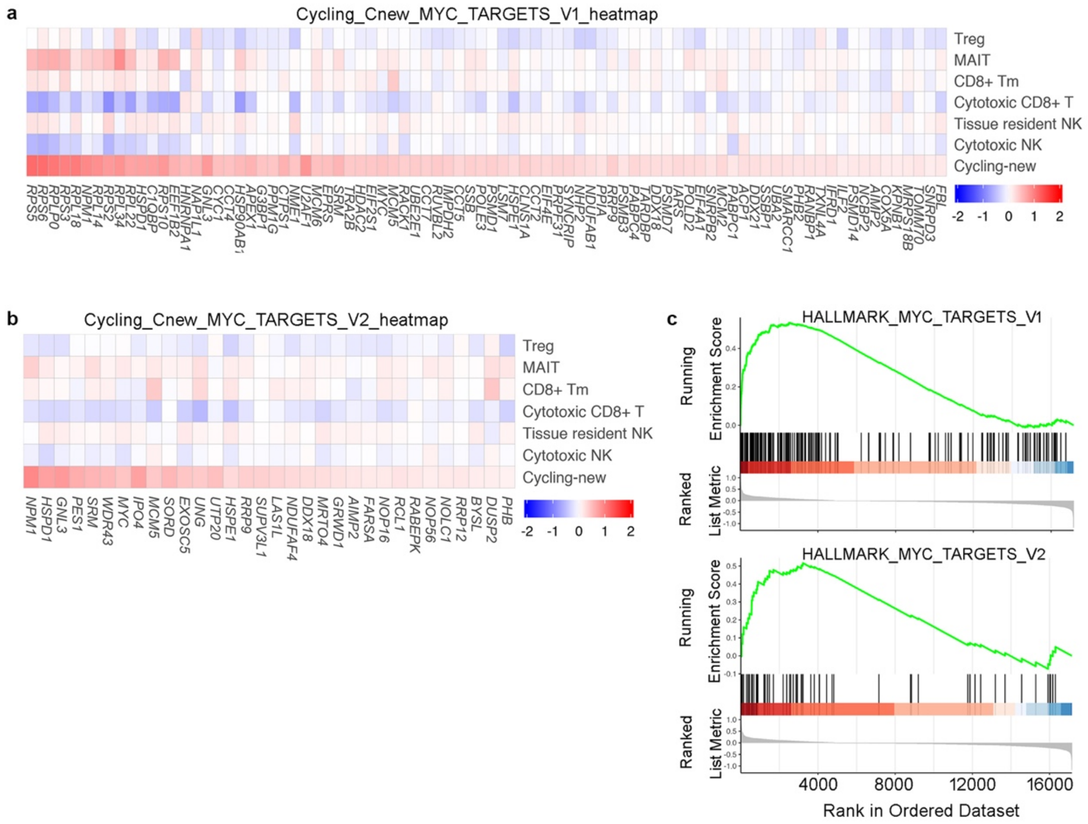

Fig. S7 The heatmap showing specific expression of Myc targeted V1 (a) and Myc targeted V2 (b) genes in C7-Cycling subsets. (c) The GSEA plot of Myc targeted V1 and V2 pathway in C7-Cycling subsets.

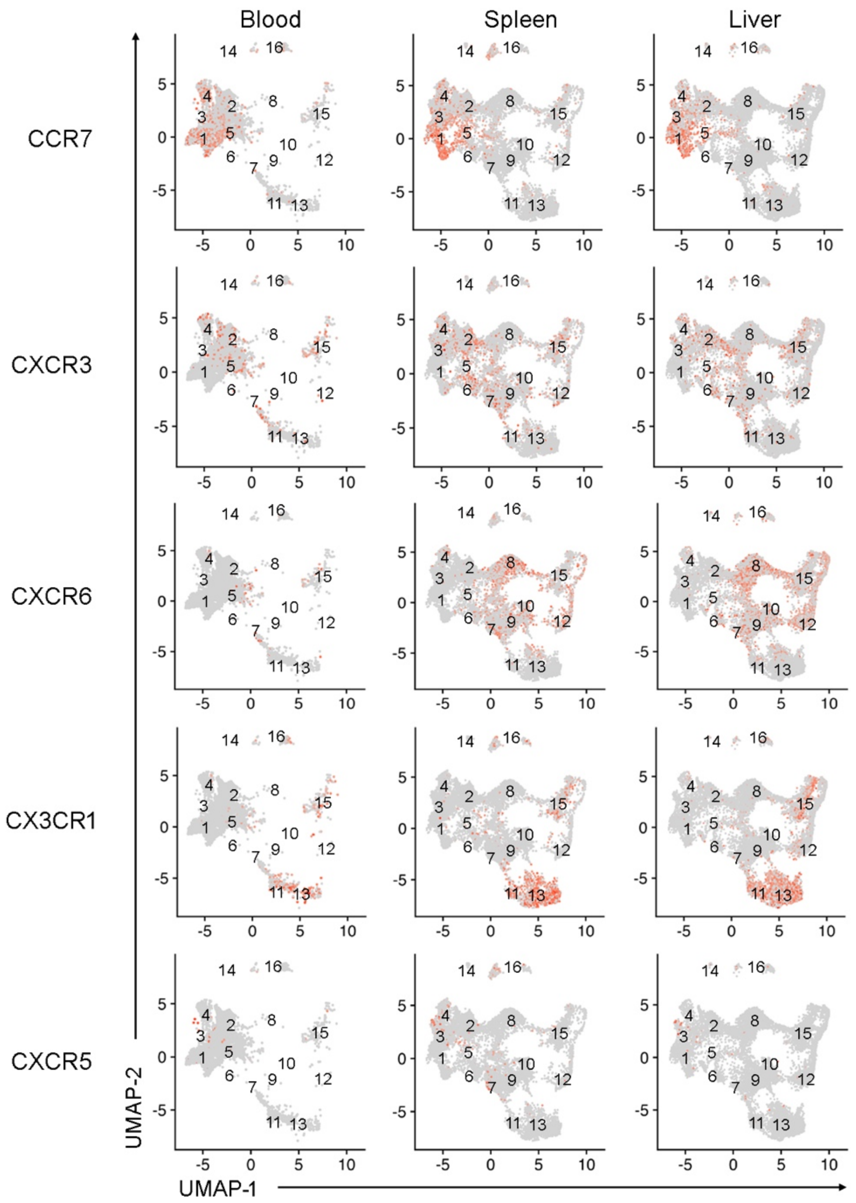

Fig. S8 UMAP showing the expression of chemokine receptors including *CCR7, CXCR3, CXCR6, CX3CR1* and *CXCR5* by various tissues.

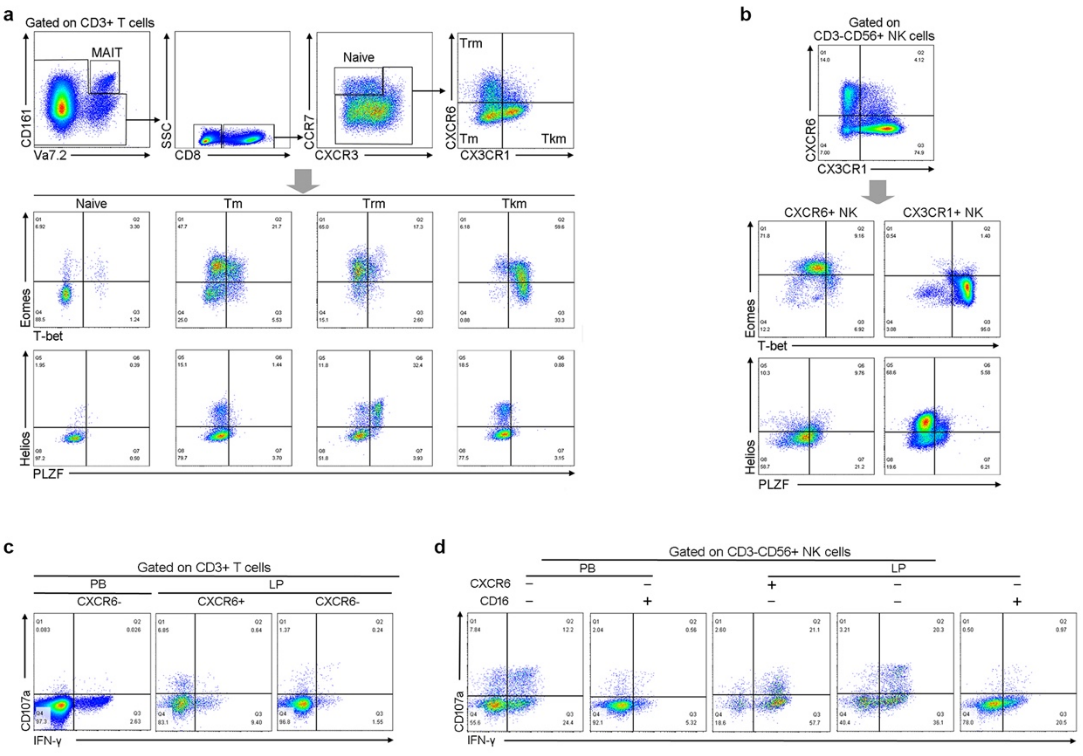

Fig. S9 The expression of transcriptional factors and functional characterization of liver resident NK&T cells. (a-b) Flow cytometry gating strategy to identify naïve/CM CD8$^+$ T cells (CCR7$^+$), memory CD8$^+$ Tm cells (CXCR3$^+$), tissue-resident memory CD8 Trm and MAIT cells (CXCR6$^+$), CD8$^+$ Tc cells (CX3CR1$^+$) (a), and liver-resident NK cells (LrNK) and cNK cells (b). The expression of four transcriptional factors T-bet, EOMES, PLZF and Helios by each CD8$^+$ T cell (a) and NK cell (b) subset were detected. (c-d) Representative dot plots showing CD107a and IFN-γ production by various T (c) and NK (d) cell subsets in responses to IL-12/IL-18 stimulation for 6 hours in vitro.

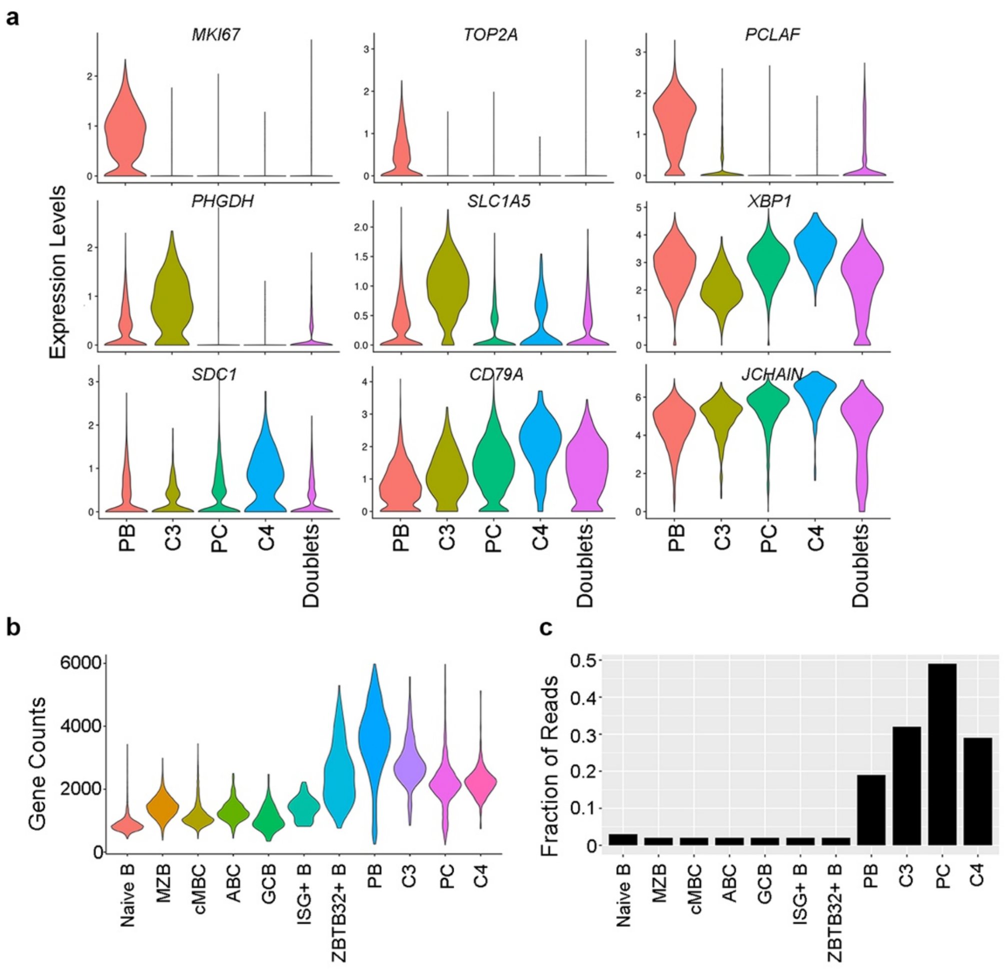

Fig. S10 Analysis of specific markers of ASC subsets. (a) Violin plots showing the expression of selected markers in various ASC subsets. (b) Gene counts of B cell and ASC subsets after exclusion of immunoglobulin genes. (c) The fraction of reads annotated as immunoglobulin genes in various B and ASC subsets.

Table S1 The basic information of donors

Table S2 Cell counts analyzed in the study.

Table S3 The differentially-expressing gene list of total T and NK cells

Table S4 The differentially-expressing gene list of liver-resident T and liver-resident NK cells

Table S5 The co-expression gene list of CXCR6^{+} CD8^{+} T cells, MAIT and CXCR6^{+} NK cells

Table S6 The differentially-expressing gene list of cycling T and NK cells

Table S7 The differentially-expressing gene list of myeloid cells

Table S8 The differentially-expressing gene list of tissue-derived monocytes vs blood-derived monocytes

Table S9 The differentially-expressing gene list of total B cells and tissue-derived cMBC and ZBTB32^{+} B vs their blood-derived counterparts.

Table S10 The differentially-expressing gene list of total ASC cells

Table S11 The specific differentially-expressing gene list of B and ASC cells

Table S1 The basic information of donors

	Donor-1	Donor-2	Donor-3
Age (years)	41	27	56
Gender (male/female)	male	male	male
DBD/DCD	DBD	DBD	DBD
Clinical factors at transplant			
Accident leading to donation	Emerging	Traffic	Traffic
Reasons leading to donation	Brain palsy	Brain palsy	Brain palsy
Days since the onset of donation accident	20	8	8
WBC ($\times 10^9$)	12.7	15.9	8.33
Red cell ($\times 10^{12}$)	2.71	3.47	2.79
Hemoglobin (g)	89	108	85
Platelet ($\times 10^9$)	291	463	193
Prothrombin time (second)	13.4	14.1	12.3
Prothrombin activity	64.8	58	78.2
Alanine aminotransferase (U/L)	95	28	26
Total bilirubin (μmol/L)	9.6	13.2	14.9
Direct bilirubin (μmol/L)	4.6	5.9	4.8
Creatinine (μmol/L)	59	67	72
Albumin (g/L)	28	25	34
C-reactive protein (mg/L)	108.9	104	102
HBsAg (+/-)	-	-	-
Anti-HCV antibody (+/-)	-	-	-

DBD, donation after brain death; DCD, donation after circulatory death; HBsAg, hepatitis B surface antigen; HCV, hepatitis C virus

Table S2 Cell counts analyzed in the study

No	Donor	Organ	Cell counts	Median gene/cell	Mean K Reads/cell	Median UMIs/cell	Fraction Reads in cells (%)	Total cell counts
J1	D1	Blood	1922	1230	126	3873	91.7	
J7	D2	Blood	8188	1477	63	4599	95.1	20182
J13	D3	Blood	10072	1056	49	3776	95.8	
J2	D1	Spleen	9050	1343	44	4761	96.2	
J8	D2	Spleen	10814	1336	46	4534	94.6	35034
J14	D3	Spleen	15170	1331	65	4823	96.0	
J3	D1	LP	7391	1468	55	4354	95.9	
J9	D2	LP	12886	1369	61	3908	94.4	32716
J15	D3	LP	12439	1127	40	3287	95.4	
Mean			9770	1304	61	4213	95.0	29311

LP, liver perfusion; D, donor
